# Isorhamnetin Alleviates Early-Life Stress-Induced Anxiety and Depression in Male Mice: Neuroinflammatory Modulation and Sirt1/NF-κB Signaling Insights

**DOI:** 10.1007/s12035-025-05483-3

**Published:** 2025-11-29

**Authors:** Esam Qnais, Omar Gammoh, Mohammad Alqudah, Manal Al-Bzour, Mohammed Wedyan, Abdelrahim Alqudah, Anwar M. Alnakhli, Sireen Abdul Rahim Shilbayeh, Alaa A. A. Aljabali, Taher Hatahet

**Affiliations:** 1 Department of Biology and Biotechnology, Faculty of Science, The Hashemite University, Zarqa, Jordan; 2 Department of Clinical Pharmacy and Pharmacy Practice, Faculty of Pharmacy, Yarmouk University, Irbid, Jordan; 3 Physiology Department, School of Medicine and Biomedical Sciences, Arabian Gulf University, Manama, Bahrain; 4 Department of Physiology and Biochemistry, Faculty of Medicine, Jordan University of Science and Technology, Irbid, Jordan; 5 Department of Chemistry, Faculty of Science, The Hashemite University, Zarqa, Jordan; 6 Department of Clinical Pharmacy and Pharmacy Practice, Faculty of Pharmaceutical Sciences, The Hashemite University, Zarqa, Jordan; 7 Department of Pharmaceutical Sciences, College of Pharmacy, Princess Nourah Bint Abdulrahman University, P.O. Box 84428, Riyadh, 11671 Saudi Arabia; 8 Department of Pharmacy Practice, College of Pharmacy, Princess Nourah Bint Abdulrahman University, P.O. Box 84428, Riyadh, 11671 Saudi Arabia; 9 Department of Pharmaceutics and Pharmaceutical Technology, Faculty of Pharmacy, Yarmouk University, Irbid, 21163 Jordan; 10 North Wales Medical School, Brigantia Building, Bangor University, Penrallt Road, Bangor, Gwynedd, Wales LL57 2AS UK; 11 Department of Medical Laboratory Sciences, Faculty of Allied Medical Sciences, Zarqa University, Zarqa, Jordan

**Keywords:** Isorhamnetin, Sirt1, Maternal separation, Anxiety, Depression, NF-kB

## Abstract

Offsprings early separated from their mothers may display depression and anxiety behaviors. The objective of this study is to assess the potential of isorhmanetin, known for its anti-inflammatory and antidepressant properties, in mitigating these behaviors in male mice. The experimental mice underwent a 4-h daily maternal separation from postnatal days 2 to 21. Afterwards, mice were given an intraperitoneal injection of isorhamnetin (20 mg/kg/day) from postnatal day 61 for 4 weeks. At 3 months old, both behavioral evaluations and biochemical analyses were carried out. The findings indicated that the isolated mice displayed clear signs of anxiety and depression-like behaviors, along with increased levels of various cytokines (IL-1β, IL-6, and TNF-α) in the hippocampus and decreased levels of Sirt1, accompanied by elevated NF-κB p65 expression. The effects were mitigated by treatment with isorhamnetin, resulting in the normalization of behavior and cytokine levels, as well as the activation of Sirt1 expression in addition to downregulation of NF-κB p65. Isorhamnetin has the potential to be utilized as a treatment for anxiety and depression-like symptoms by adjusting pathways of neuroinflammation and the signaling axis of Sirt1 and NF-κB. This opens the door for further investigation in mitigating the biochemical and behavioral adverse effects induced by separation during the maternal period.

**Supplementary Information:**

The online version contains supplementary material available at 10.1007/s12035-025-05483-3.

## Introduction

Over the last few decades, studies have brought more attention to the crucial impact of stressors in early life, such as the event of being separated from the mother, in predisposing individuals to psychiatric disorders later in life [1, 2]. These early events are believed to trigger a cascade of neurobiological changes in the hippocampus, a region integral to emotional regulation and memory processing. The elevated density of glucocorticoid receptors in the hippocampus mediates responses to stress by influencing gene expression and neuronal function [3, 4].

Maternal separation (MS) has been extensively employed as a model to simulate early-life stress in rodents [5]. This model has consistently demonstrated that prolonged separation from the mother induces marked behavioral changes in offspring, such as increased anxiety and depressive-like symptoms, which are often persistent into adulthood. These behavioral changes are closely linked to alterations in the neuroimmune environment of the hippocampus [6]. Studies have shown that cytokines, including IL-1β, TNF-α, and IL-6, are elevated, alongside changes in the activity of microglial cells, the resident immune cells of the brain [7–9]. These neuroimmune responses can alter neuronal plasticity and contribute to the pathophysiology of depression and anxiety disorders [10], which further highlights the significance of the hippocampal neuroimmune axis in the context of early-life stress. Moreover, the long-term effects of early-life stress on neurodevelopment and behavior include significant alterations in brain structure and function. This consequently predisposes individuals to a range of psychiatric conditions.

Emerging research suggests that phytochemicals may have the potential to modulate these neuroimmune responses, thereby mitigating the adverse effects of MS. For instance, auraptene, derived from citrus peels, has been shown to confer protective effects on both behavioral and hippocampal changes induced by MS stress, potentially through its anti-inflammatory and antioxidant properties [11]. Similarly, anethole, a pure compound commercially purchased, has demonstrated antidepressant-like effects in MS models by reducing oxidative stress and correcting nitrite imbalances within the brain [12]. This suggests a crucial role of inflammation and oxidative stress pathways in mediating stress-related behavioral outcomes. Furthermore, progesterone has been identified as a modulator of the neuroimmune response, exhibiting antidepressant-like effects in MS models by dampening neuroinflammatory responses and reducing oxidative stress in the hippocampus [13]. These studies not only reinforce the significance of the hippocampal neuroimmune axis in the context of early-life stress but also highlight novel therapeutic targets for intervention.

Isorhamnetin, extracted from the fruits of *Hippophae rhamnoides* L. and the leaves of *Ginkgo biloba* L., is recognized for its diverse pharmacological activities. These include anti-inflammatory [14], antitumor [15], antioxidant [16], brain-protective [17], memory-enhancing [18], and antidepressant properties [19]. Evidence suggests that isorhamnetin may activate Nrf2 and its target proteins, including BDNF and HO-1 while regulating the levels of NO and IL-6 [20]. Notably, it has been found to protect human retinal pigment epithelial (RPE) cells against oxidative stress and cell death induced by H_2_O_2_ by activating the PI3K/Akt signaling pathway [21]. Moreover, isorhamnetin demonstrates significant improvements in cognitive impairments caused by a high-fat and high-fructose diet (HFFD). This effect is attributed to its ability to attenuate HFFD-induced microglial activation and the release of inflammatory cytokines by inhibiting the MAPK and NF-κB signaling pathways [22]. Additionally, isorhamnetin alleviates high glucose-induced oxygen–glucose deprivation and reoxygenation-induced apoptosis, inflammatory response, and oxidative stress in HT22 cells through the Akt/Sirt1/Nrf2/HO-1 pathway [23]. Furthermore, in a study on depression induced by weightlessness using the hindlimb unloading rat model, isorhamnetin treatment significantly reduced immobility time in forced swimming and tail suspension tests [24]. The content of serotonin and dopamine in the hippocampus, which was reduced in hindlimb-unloaded rats, was increased after isorhamnetin treatment. These findings highlight the potential therapeutic effect of isorhamnetin in addressing depressive symptoms associated with weightlessness-induced conditions.

Previous studies, such as Ekici et al. [25], have effectively demonstrated the anxiolytic and antidepressant effects of isorhamnetin using acute models of neuroinflammation, focusing mainly on the biochemical responses driven by LPS and lacking long-term stress consequences. Earlier studies have highlighted the ability of isorhamnetin to regulate multiple inflammatory mediators (i.e., TNF-α, IL-1β, and IL-6) and neurotrophic factors (i.e., BDNF) within brain areas critical for mood regulation. Nevertheless, these studies only focused on acute inflammatory triggers rather than chronic stress models that are highly relevant to human psychiatric disorders. In contrast to Ekici et al. [25], who studied isorhamnetin in neuroinflammation induced by acute LPS, this study employs a model of chronic stress MS, which more realistically models early life adversity in humans. This paradigm results in enduring behavioral and neurobiological effects, including chronic cytokine dysregulation and impairment of Sirt1/NF-κB signaling in adulthood. The chronicity of stress in this study has significant translational implications. This work is unique in that it shows isorhamnetin can attenuate the lifelong neuroinflammatory and behavioral consequences of early-life stress. Therefore, this study extends the therapeutic usage of isorhamnetin from the acute inflammatory models into a developmental stress context and provides new evidence that isorhamnetin is promising for the treatment of stress-related psychiatric disorders with developmental origins.

Our study extends these findings to explore the therapeutic potential of isorhamnetin in a mouse model of chronic stress, namely, MS. As such, the model of MS has been well-validated as a model to study the long-term effects of stress in young life on adult behavior and neurobiology. It provides striking similarities to the developmental pathways that increase the risk for psychiatric illness in human populations.

For this purpose, the current study focuses on the mechanistic aspect through which isorhamnetin can attenuate the long-lasting adverse effects of early-life stress. It specifically examines the interaction of isorhamnetin with molecular signaling pathways that are already known to be dysregulated in stress-related diseases such as the Sirt1/NF-κB signaling pathway, a central axis involved in modulating neuroinflammatory responses and neural plasticity. Through analyzing these interactions, this study sheds more light on the molecular mechanisms that support isorhamnetin’s prevention of the numerous behavioral and biochemical changes induced by chronic stress.

This mechanistic exploration is crucial not only for elucidating the potential of isorhamnetin as a therapeutic agent but also for advancing our understanding of the biochemical landscape that shapes stress-related psychiatric disorders. These molecular pathways can be targeted to develop new strategies to treat psychiatric disorders resulting from chronic stress. As a result, our study is a valuable addition to the fields of neuropharmacology and mental health.

## Materials and Methods

### Animals

Ten-week-old Swiss albino male and female mice with an average weight of 22–25 g were obtained from the animal facility at the Hashemite University in Zarqa, Jordan. They were placed in a regulated environment where the temperature was maintained at 23 ± 1 °C and 56 ± 5% humidity. A 12-h light and dark cycle starting at 07:00 am was also employed. The mice were allowed to access food and water freely during a 2-week acclimatization period. The breeding process started by matching two females with one male. Postnatal day 0 (PND 0) was considered the day of birth, and the study primarily concentrated on male offspring.

#### Separation Procedure

The young pups were split into two sets. The initial set experienced maternal separation (MS), which involved removing the pups from their mothers for 4 h each day from 10:00 to 14:00, commencing on the second day after their birth (PND 2) and lasting until the twenty-first day (PND 21) following the procedure described by Huang et al. [26]. The pups that were separated were placed in clean cages with bedding on a warm pad. Another group of pups did not experience maternal separation (NMS) and stayed with their mothers constantly until they were weaned.

#### Experimental Groups

**Fig. 1:**
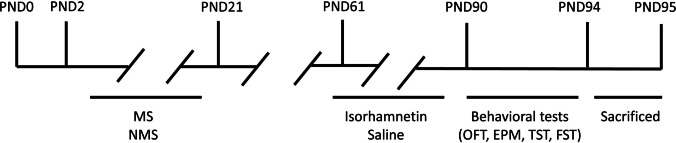
Experimental flowchart

The research involved 32 mice, divided into four groups of eight mice each. To specifically study the impact of MS stress on male mice, we focused only on male subjects. The mice were separated into four groups: a control group (CON), a second group that received isorhamnetin treatment (the control group received isorhamnetin), a group that underwent maternal separation (MS), and a group that was treated with isorhamnetin (the separated group was treated with isorhamnetin). For 4 weeks, mice in the experimental groups were administered isorhamnetin injections at a daily dosage of 20 mg/kg. Isorhamnetin, a yellow crystalline compound with a purity level exceeding 98%, was dissolved in saline (90%), DMSO (5%), and Tween 80 (5%). The CON and MS groups received injections of a saline solution containing 5% DMSO and 5% Tween 80 but did not include isorhamnetin (Fig. 1). Behavioral evaluations were carried out without prior knowledge of the group allocation for each animal. The sample size was determined based on previous experimental results [27]. We selected the 20 mg/kg/day dosage based on preliminary studies that evaluated various doses of isorhamnetin for their effectiveness in alleviating depressive-like symptoms in both normal and maternally separated mice. The effectiveness was lower with doses under 10 mg/kg, while doses above 20 mg/kg did not enhance the benefits of isorhamnetin treatment and increased the risk of toxicity. The 20 mg/kg dose was selected due to its optimal combination of effectiveness and safety. This dosage was exclusively used in the main study to focus on the therapeutic effects of isorhamnetin without the confusion of multiple dose levels.

The treatment duration of 4 weeks was chosen to provide ample time for isorhamnetin to have its pharmacological impact on neuroimmune pathways and behavioral reactions. This period aligns with the timelines used in similar preclinical studies, where chronic treatment periods are typically required to observe significant changes in behavioral and biochemical markers of psychiatric disorders [28, 29].

#### Open-Field Test

The open-field test was used to assess behaviors similar to anxiety on postnatal day 91 (PND 91) in mice, following the protocol outlined previously. This assessment quantifies anxiety levels based on the mice’s avoidance of the central zone within an open field. The testing area consisted of a 50 cm × 50 cm square enclosure with 25-cm-high walls, illuminated at 30 lx. The center of the arena was where each mouse was placed for 5 min to explore freely. Between trials, 75% alcohol was used to clean the arena and left to air-dry, eliminating the remaining scents from previous assessments. Parameters recorded included the time spent, the number of entries, and the distance traveled in the central area (in total) during the 5 min. 

#### Elevated-Plus Maze Test

The elevated-plus maze test was conducted on PND 92, following the method described by Wei et al. [27]. The purpose of this assessment is to measure anxiety-related behaviors in mice. The reduced time spent in the open arms associated with reduced entry numbers are indicators of heightened anxiety levels. The maze used was in the shape of a cross and was illuminated at 30 lx. Two arms (open) and two arms (enclosed) were arranged in opposite positions. Arms measured 30 cm (length) and 6 cm (width), while the arms that were enclosed had a 15-cm height. The entire structure was raised 80 cm above the ground and included a central square measuring 6 cm × 6 cm. The central square contained mice that were given 6 min to explore while facing an open arm freely. To avoid scent bias, 75% alcohol was used to clean the maze before each test. The time spent by each mouse and the frequency of their entries into open arms were recorded.

#### Tail-Suspension Test

The tail-suspension test was conducted on PND 93 to assess depression-like behaviors in mice [30]. To perform the assessment, we used a tape to fasten the tail of each mouse (1 cm from the end). After that, the mouse was hung by this taped area from a hook, with its nose positioned above the ground (around 35 cm). A cylinder (plastic) was used to encase the tail to prevent the climbing of the mouse. The behavior of every mouse was recorded for 6 min, with only the last 4 min of this footage being analyzed. During these 4 min, an observer, without knowledge of the mice’s group assignments, measured the duration of immobility to evaluate depression-like behaviors quantitatively. Higher levels of depressive behavior are indicated by more extended periods of immobility in this test.

#### Forced Swimming Test

On the day after the tail suspension test, which was on postnatal day 94, we performed the forced swimming test on the mice to evaluate their depression-like behaviors. For this experiment, we employed a transparent glass tube measuring 28 cm (height) and 18 cm (width). The tube contained water at a temperature of 22 ± 1 °C, and the water level was at 15 cm. Every mouse was submerged in the water for the full 6-min duration of the trial. The entire process was documented for later examination. An observer, who was not informed of the group assignments, reviewed the last 4 min of the recording to measure the duration of time each mouse spent immobile. The mouse is considered immobile when it is either floating passively or making minimal movements to maintain balance. By observing their immobility during the forced swim, behaviors similar to those observed in depression can be assessed.

To avoid carryover effects and reduce cumulative stress, all behavioral tests were conducted at least 24 h apart, with a single test administered per day. This approach follows established protocols in rodent behavioral research—for example, scheduling separate assessment days for the Elevated Plus Maze, Tail Suspension Test, and Forced Swim Test [31, 32].

#### Tissue Preparation

The mice were euthanized at postnatal day 95 by administering a 2% sodium pentobarbital solution. After euthanizing the mice, each hippocampus was excised bilaterally. Immunoblotting and ELISA were used to measure cytokine levels and protein levels, respectively. RNA was extracted from the contralateral hippocampus by RT-qPCR analysis. This allowed us to carry out protein and gene expression analyses using different samples, preventing any cross-contamination and optimizing the integrity of outcomes. Subsequently, the tissue was frozen at − 80 °C for extended preservation and subsequently utilized for biochemical analyses to verify its appropriateness for detailed scientific investigation.

Euthanasia was performed by a 2% solution of sodium pentobarbital (intraperitoneal, 100 mg/kg body weight). Based on the rapid and humane induction of death while minimizing suffering and stress to the animal (consistent with the guidelines outlined by the US National Institutes of Health regarding the use and care of laboratory animals [33]).

Pentobarbital, a barbiturate, functions to induce anesthesia followed by euthanasia by depressing central nervous system functions that suppress respiration and cardiac function [34]. The choice of pentobarbital was also made with consideration for its minimal influence on the necessary molecular targets, specifically cytokines and neuroinflammatory markers [35–37]. However, to ensure the reliability of our results, the procedure was applied uniformly to all experimental groups, and animals were euthanized at the same time point relative to the last treatment to standardize the procedure.

#### Measurement of Cytokine Levels

The mixed samples underwent centrifugation at speeds ranging from 2000 to 3000 rpm for 20 min to separate the clear liquids on top. For further analysis, these liquids were obtained carefully. The levels of cytokines (IL-1β, IL-6, TNF-α) in mice were determined using specific ELISA kits from MyBiosource, USA. The kits have the following sensitivities or limits of detection: IL-1β, 1 pg/mL; IL-6, 1 pg/mL; TNF-α, 1.2 pg/mL. Sample dilutions were optimized during preliminary experiments to fall within the dynamic range of the standard curve, typically at 1:2 for serum samples. The collected liquids were used to determine these cytokine levels according to the manufacturer’s instructions. The measurement of cytokine levels relied on optical density readings, and eight samples from each group were consistently analyzed using this standardized method to ensure reliable results.

#### Real-Time PCR Relies on Fluorescence for Quantitative Measurements

The TRIzol (Thermo Fisher Scientific, Cat# 15,596,026) method was used as previously described [38]. Subsequently, a total of 1 µg of total RNA was reverse transcribed using a standard cDNA synthesis kit (Applied Biosystems, Cat# 4,368,814) according to the manufacturer’s protocol. This amount of RNA is sufficient to generate a comprehensive cDNA library representing the gene expression profile of the sample. For each real-time PCR reaction, 1 µL of the resulting cDNA was used as the template. This quantity is typically sufficient to quantify gene expression levels across multiple targets without prematurely depleting the cDNA resource. In our RT-qPCR assays, 1 µL of each primer (forward and reverse) from a 10 µM stock solution was used, resulting in a final primer concentration of approximately 400 nM per reaction. This concentration is commonly used in quantitative PCR to ensure optimal binding efficiency and specificity. Table 1 represents the primers that were utilized for qPCR to amplify the target genes (NF-κB p65, Sirt1, β-actin). β-actin was chosen due to its stable expression in the mouse hippocampus across various experimental conditions, making it an appropriate internal control for normalizing gene expression data. Its use in RT-qPCR ensures consistency and reliability in measuring gene expression changes, particularly in studies involving neural tissue [39]. The reaction mixture for PCR comprised 5 µL of 2 × SYBR Green Mix (Applied Biosystems, Cat# 4,309,155), 1 µL each of forward and reverse primers, 1 µL of cDNA, and 2 µL of RNase-free water. Initial denaturation at 95 °C for 60 s was performed as the first step of the thermal cycling process, 40 cycles at 95 °C (20 s) was the second step, and finally, at 60 °C (60 s). The 2^ − ΔCt technique was used to evaluate gene expression levels. In this technique, ΔCt represents the disparity between the target and reference genes at the cycle threshold (Ct). The development of this method can be attributed to Livak and Schmittgen in 2001 [40].

**Table 1:** The sequences of the primers used for qPCR

Gene	Amplicon size (bp)	Forward primer (5′ → 3′)	Reverse primer (5′ → 3′)
β-actin	120	AGTGTGACGTTGACATCCGT	TGCTAGGAGCCAGAGCAGTA
NF-kB p65	119	GCTCCTGTTCGAGTCTCCAT	TTGCGCTTCTCTTCAATCCG
Sirt1	116	TAATGTGAGGAGTCAGCACC	GCCTGTTTGGACATTACCAC

#### Western Blotting

The extraction of proteins from the hippocampal tissue involved the use of RIPA lysis buffer (Sigma-Aldrich, Cat# R0278), followed by centrifugation (12,000 g for 15 min) to collect the supernatant. Protein concentrations in the samples were determined using the BCA Protein Assay Kit (Pierce, Cat# 23,225). Based on this quantification, equal amounts of protein (30 µg per well) were loaded to ensure comparability across samples. Following this, the proteins were combined with SDS–PAGE (5x) loading buffer (1:4 ratio) and then heated (15 min) to ensure that denaturation was complete before being prepared for gel electrophoresis. Subsequently, SDS-PAGE was carried out at 80 V for 1 h, and the proteins were transferred onto membranes (PVDF) (Millipore Sigma, Cat# IPVH00010). The transfer times were adjusted for each protein, allotted for NF-kB p65 and its acetylated form (50 min) and for Sirt1 (70 min). Following this, the PVDF underwent a washing and blocking procedure using 5% skim milk powder for a duration of 2 h at room temperature for nonspecific binding prevention. Membranes were subsequently exposed to primary antibodies against Sirt1 (Cell Signaling Technology, Cat# 9475, 1:1000 dilution), NF-kB p65 (Abcam, Cat# ab16502, 1:1000 dilution), and its acetylated form (Cell Signaling Technology, Cat# 3045, 1:500 dilution). Primary antibodies were diluted correctly and left overnight (at 4 °C). The membranes underwent incubation with Goat anti-rabbit IgG (H + L) secondary antibody, HRP conjugate (Bio-Rad, Cat# 170–6515, 1:2000 dilution), or Goat anti-mouse IgG (H + L) secondary antibody, HRP conjugate antibodies (Bio-Rad, Cat# 170–6516, 1:2000 dilution) for 1.2 h at room temperature. The detection of protein bands was carried out using an ECL ultrasensitive kit (Thermo Fisher Scientific, Cat# 34,096), and quantification was performed using ImageJ software. GAPDH was employed as the reference control to ensure the accurate assessment of protein expression levels by normalizing the target protein gray values.

#### Statistical Analysis

All statistical analyses were performed using GraphPad Prism software (Version 8.0). Normality and homogeneity of variances were examined using the Shapiro–Wilk and Levene’s tests, respectively, before conducting the parametric tests. All datasets met these assumptions (*p* > 0.05), and a two-way ANOVA was employed. This analysis was performed to characterize the impact of two experimental conditions (maternal separation vs. no maternal separation) and two treatments (isorhamnetin vs. saline). Tukey’s post hoc multiple comparisons test was performed for the multiple-way ANOVA when significant main and interaction effects were found. Results are expressed as the mean ± SEM, and *p* < 0.05 was considered significant.

## Results

### Isorhamnetin Mitigated Anxiety-Like Behaviors Induced by Maternal Separation

**Fig. 2:**
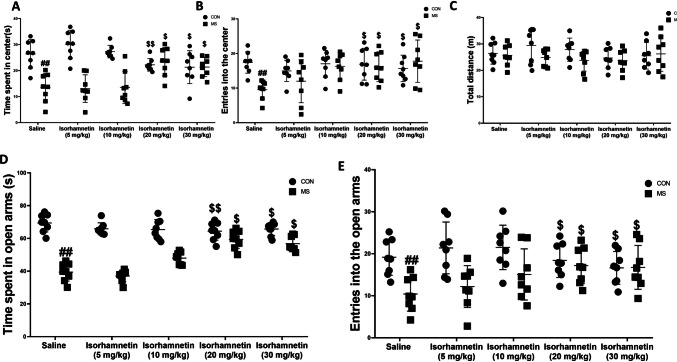
Isorhamnetin effectively mitigated anxiety-like behaviors induced by maternal separation in mice, as assessed through the open-field test (**A**–**C**) and the elevated plus maze test (**D**, **E**). Specifically, the evaluation included measuring **A** the time spent in the central region during the open-field test, **B** the number of entries into the central region during the open-field test, and **C** the total distance covered in the open-field test. In the elevated plus maze test, **D** the time spent in the open arms and **E** the number of entries into the open arms were documented. All presented data represent mean values ± SEM. Significant comparisons are highlighted as follows: saline-treated MS group vs. saline-treated control group (##*p* < 0.01), isorhamnetin-treated control group vs. saline-treated control group (*p* > 0.05), and isorhamnetin-treated MS group vs. saline-treated MS group ($*p* < 0.05, $$*p* < 0.01). The study encompassed eight subjects per group, with CON referring to the control group and MS indicating the maternal separation group

The research focused on examining how isorhamnetin affects anxiety-related behaviors in male offspring subjected to maternal separation. The open-field test results were evaluated using a two-way ANOVA. It revealed significant effects of experimental condition (maternal separation vs. no maternal separation) and treatment group (isorhamnetin vs. saline) (*F*(1, 28) = 6.35, *p* = 0.017) and a significant interaction between treatment and isorhamnetin (*F*(1, 28) = 4.88, *p* = 0.035). However, isorhamnetin alone did not significantly influence the time spent in or entries into the central area (*p* = 0.632), as depicted in Fig. 2A, B. Maternal separation significantly decreased the duration of time spent in and entries into the central zone compared to the control group treated with saline (*F*(1, 28) = 12.04, *p* = 0.001). Still, isorhamnetin treatment effectively countered these effects (*F*(1, 28) = 5.47, *p* = 0.026). No significant differences were observed between the isorhamnetin-treated control group and the saline-treated control group in terms of time spent and entries (*F*(1, 28) = 0.98, *p* = 0.332). The distance traveled within 5 min did not differ significantly across groups (*F*(1, 28) = 0.52, *p* = 0.475), as illustrated in Fig. 2C.

The time spent and frequency of entries into the open arms, from the elevated plus maze assessment, indicated significant effects from the experimental condition (maternal separation vs. no maternal separation) (*F*(1, 28) = 7.32, *p* = 0.011). Similarly, the interaction between experimental condition and treatment group was significant (isorhamnetin vs. placebo) (*F*(1, 28) = 6.03, *p* = 0.020) on without any considerable influence from isorhamnetin alone (*p* = 0.590) (Fig. 2D, E). The MS group showed a decline in both the duration (*F*(1, 28) = 13.47, *p* = 0.0008) and number of entries (*F*(1, 28) = 11.89, *p* = 0.0014) into the open arms compared to the control group treated with saline. However, these decreases were alleviated by isorhamnetin treatment (duration: *F*(1, 28) = 4.22, *p* = 0.049; entries: *F*(1, 28) = 4.78, *p* = 0.037). No significant differences were observed between the isorhamnetin-treated control group and the saline-treated control group in terms of duration (*F*(1, 28) = 0.39, *p* = 0.537) and entries (*F*(1, 28) = 0.54, *p* = 0.467).

#### Isorhamnetin Alleviated Symptoms of Depression in Male Offspring Caused by Separation from Their Mothers

The research demonstrated that isorhamnetin effectively decreased symptoms of depression in male offspring who experienced MS, as evidenced by tail suspension and forced swimming tests.

**Fig. 3:**
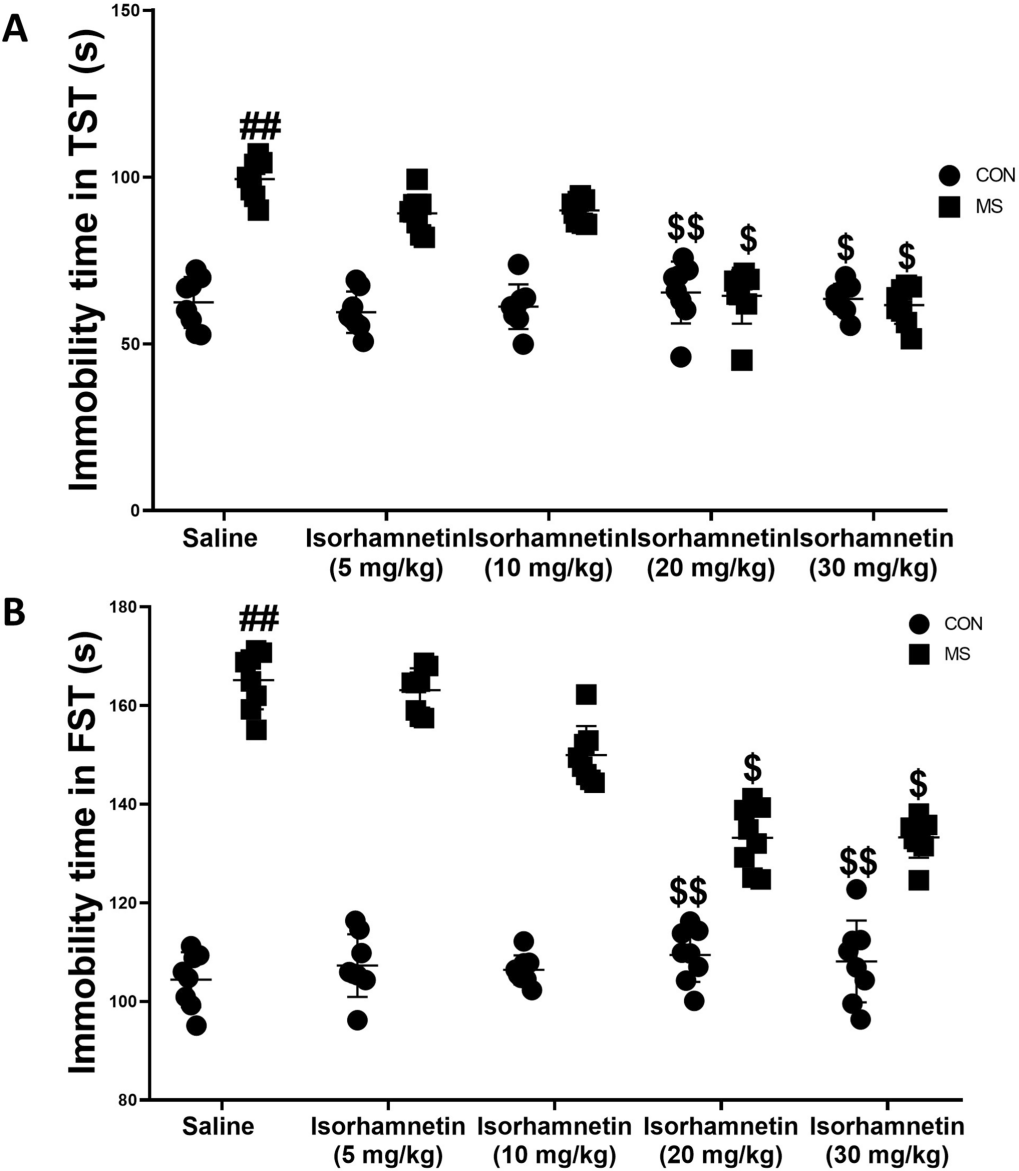
Isorhamnetin mitigated depression-like behaviors induced by maternal separation in mice. The assessment of depression-like behavior involved the tail suspension and forced swimming tests, with a specific focus on measuring **A** immobility time in the tail suspension test and **B** immobility time in the forced swimming test. The data are presented as means ± SEM. Significant comparisons are highlighted as follows: saline-treated MS group vs. saline-treated control group (##*p* < 0.01), isorhamnetin-treated control group vs. saline-treated control group (*p* > 0.05), and isorhamnetin-treated MS group vs. saline-treated MS group ($*p* < 0.05, $$*p* < 0.01). The study included eight subjects per group, with CON signifying the control group and MS representing the maternal separation group

In the tail suspension test, significant differences were observed between maternal separation vs. no maternal separation (*F*(1, 28) = 5.93, *p* = 0.021) and between isorhamnetin vs. saline (*F*(1, 28) = 4.06, *p* = 0.053). Isorhamnetin alone did not significantly change immobility times (*F*(1, 28) = 2.34, *p* = 0.137), as shown in Fig. 3A. The group that underwent MS exhibited significantly more extended periods of immobility compared to both the saline-treated control group (*F*(1, 28) = 10.47, *p* = 0.003) and the group that experienced MS but was treated with isorhamnetin (*F*(1, 28) = 6.58, *p* = 0.016). However, the differences in immobility times between the MS group, isorhamnetin group, and the saline-treated control group were not statistically significant (*F*(1, 28) = 1.09, *p* = 0.305).

Similarly, the forced swimming test, as shown in Fig. 3B, indicated a significant effect of the maternal separation vs. no maternal separation on immobility times (*F*(1, 28) = 7.22, *p* = 0.012). However, no significant interaction effect was observed between the isorhamnetin treatment group vs. saline (*F*(1, 28) = 0.98, *p* = 0.331). Further examination revealed that the group subjected to maternal separation exhibited more extended periods of immobility compared to both the control group treated with saline (*F*(1, 28) = 12.35, *p* = 0.001) and the MS group treated with isorhamnetin (*F*(1, 28) = 5.04, *p* = 0.032). This suggests that the administration of isorhamnetin alleviated the depression-like behaviors caused by maternal separation.

#### Isorhamnetin Lowered the High Levels of Cytokines Associated with Maternal Separation

**Fig. 4:**
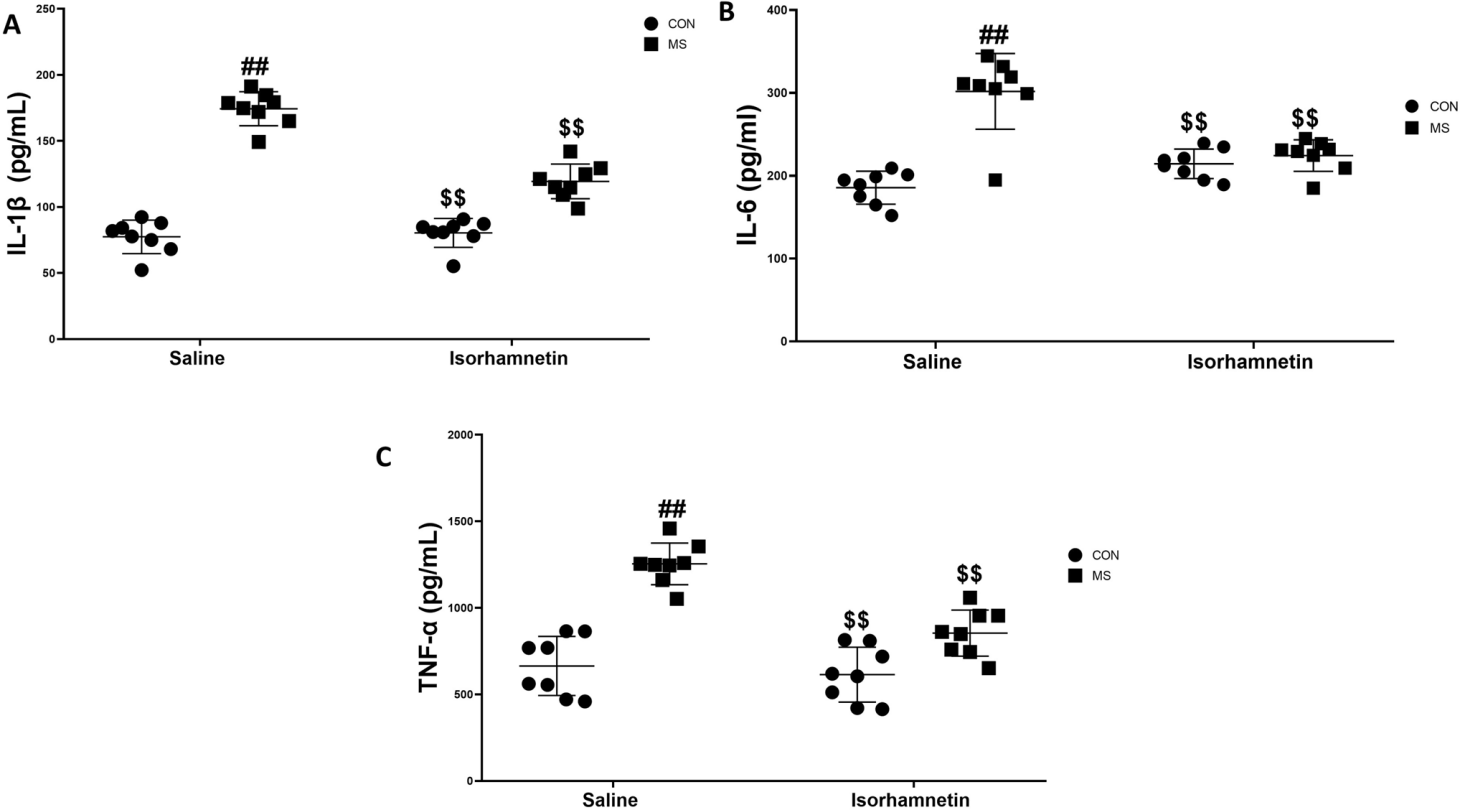
Isorhamnetin effectively suppressed the maternal separation-induced elevation in the levels of IL-1β, IL-6, and TNF-α in the mouse hippocampus. Specific measurements for **A** IL-1β, **B** IL-6, and **C** TNF-α were conducted. All data are presented as means ± SEM. Significant comparisons are highlighted as follows: saline-treated MS group vs. saline-treated control group (##*p* < 0.01), isorhamnetin-treated control group vs. saline-treated control group (*p* > 0.05), and isorhamnetin-treated MS group vs. saline-treated MS group ($$*p* < 0.01). The study comprised eight subjects per group, with CON signifying the control group and MS indicating the maternal separation group

Our findings demonstrate that isorhamnetin significantly decreases the heightened levels of pro-inflammatory cytokines in the hippocampus that result from MS. This impact was particularly pronounced in the levels of IL-1β, IL-6, and TNF-α. The administration of isorhamnetin resulted in statistically significant reductions in these cytokines, as illustrated in Fig. 4A–C.

A two-way ANOVA showed that the experimental condition of MS resulted in significantly higher cytokine levels compared to the groups that did not undergo MS and was treated with saline (IL-1β: *F*(1, 28) = 14.36, *p* = 0.0006; IL-6: *F*(1, 28) = 12.21, *p* = 0.0012; TNF-α: *F*(1, 28) = 13.47, *p* = 0.0008). The introduction of isorhamnetin significantly reduced these heightened levels (IL-1β: *F*(1, 28) = 11.58, *p* = 0.0018; IL-6: *F*(1, 28) = 10.34, *p* = 0.0034; TNF-α: *F*(1, 28) = 11.02, *p* = 0.0021), underscoring its therapeutic potential.

Additionally, no significant variations were observed in the concentrations of IL-1β, IL-6, and TNF-α between the control group treated with saline and the control group treated with isorhamnetin. This suggests that isorhamnetin had no substantial impact on cytokine levels in the absence of MS stress (IL-1β: *F*(1, 28) = 0.35, *p* = 0.558; IL-6: *F*(1, 28) = 0.42, *p* = 0.520; TNF-α: *F*(1, 28) = 0.39, *p* = 0.536). These findings suggest that the beneficial impact of isorhamnetin on cytokine levels is most prominent under conditions of stress, such as those induced by MS.

#### Isorhamnetin Lowered Sirt1 and NF-kB p65 mRNA Levels in the Hippocampus

**Fig. 5:**
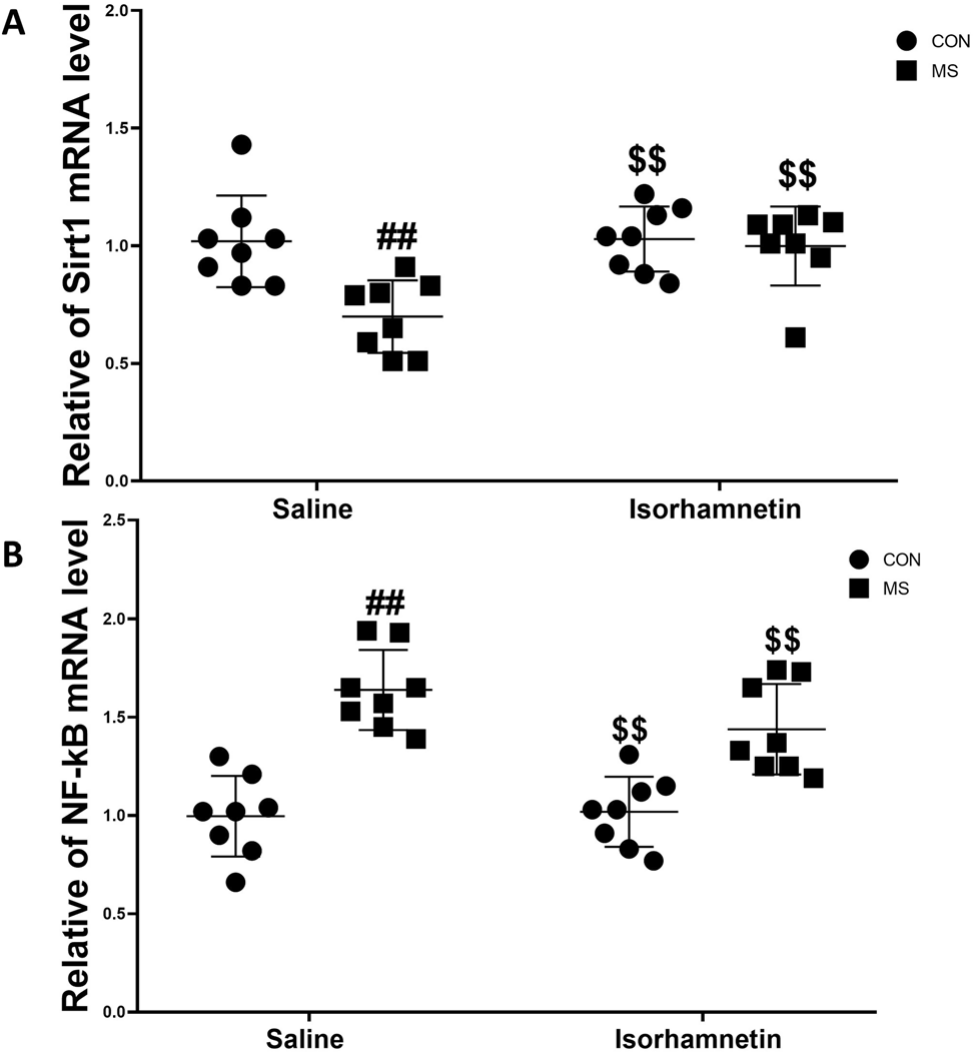
Isorhamnetin effectively reversed the maternal separation-induced decrease in Sirt1 mRNA expression levels and the concurrent increase in NF-kB p65 mRNA expression levels within the hippocampus. The specific assessments for **A** relative Sirt1 mRNA expression levels and **B** relative NF-kB p65 mRNA expression levels were conducted. All data are presented as means ± SEM. Significant comparisons are highlighted as follows: saline-treated MS group vs. saline-treated control group (##*p* < 0.01), isorhamnetin-treated control group vs. saline-treated control group (*p* > 0.05), and isorhamnetin-treated MS group vs. saline-treated MS group ($$*p* < 0.01). The study included eight subjects per group, with CON denoting the control group and MS indicating the maternal separation group

The study effectively demonstrated the impact of isorhamnetin on changing the mRNA expression levels of Sirt1 and NF-kB p65 in the hippocampus of mice exposed to MS, as depicted in Fig. 5. A two-way ANOVA revealed significant differences between MS vs. NMS and isorhamnetin vs. placebo, as well as their combined effect on gene expression. Detailed post hoc analysis indicated significant regulatory changes. Specifically, MS group exhibited a substantial reduction in Sirt1 mRNA levels compared to the control group treated with saline, showing an apparent decrease (*F*(1, 28) = 13.42, *p* = 0.0007). In contrast, the group that experienced MS and received isorhamnetin treatment demonstrated a notable increase in Sirt1 mRNA levels compared to those treated with saline alone (*F*(1, 28) = 12.35, *p* = 0.0011).

Regarding NF-kB p65 mRNA levels, there was a significant elevation in the MS group as compared to the control group treated with saline (*F*(1, 28) = 14.58, *p* = 0.0005). However, isorhamnetin treatment significantly reduced these levels in the MS group, indicating effective modulation (*F*(1, 28) = 13.67, *p* = 0.0009). Between the control groups, there were no significant differences in the mRNA levels of Sirt1 and NF-kB p65, suggesting that isorhamnetin did not affect these gene expressions in the absence of stress from maternal separation (Sirt1: *F*(1, 28) = 0.86, *p* = 0.361; NF-kB p65: *F*(1, 28) = 1.03, *p* = 0.318).

These findings highlight the therapeutic potential of isorhamnetin in reversing stress-related changes in gene expression induced by MS. The normalization of Sirt1 and modulation of NF-kB p65 mRNA levels to those closer to the control group highlight isorhamnetin’s effectiveness in modulating gene expression profiles associated with stress responses in the hippocampus.

#### Isorhamnetin Modulates Protein Levels of Sirt1, NF-kB p65, and Acetyl-NF-kB p65 Proteins in the Hippocampus

The study focused on the effects of isorhamnetin on protein expression in the hippocampus, particularly examining changes induced by MS. Our findings demonstrated significant effects of isorhamnetin on the protein levels of Sirt1, NF-κB p65, and acetyl-NF-κB p65. Statistical analysis using a two-way ANOVA revealed significant changes in protein expression induced by MS. Specifically, the MS group exhibited an increase in the protein levels of NF-kB p65 and acetyl-NF-kB p65, as well as a decrease in Sirt1 levels compared to the saline-treated control group (NF-kB p65: *F*(1, 28) = 14.25, *p* = 0.0005; acetyl-NF-kB p65: *F*(1, 28) = 13.67, *p* = 0.0007; Sirt1: *F*(1, 28) = 12.98, *p* = 0.0009).

These results suggest that MS stress leads to alterations in protein expression associated with inflammatory and stress-response pathways. Notably, the protein levels of NF-kB p65 and acetyl-NF-kB p65 decreased significantly in mice that underwent MS but received isorhamnetin treatment, while their Sirt1 levels increased, showing a marked improvement (NF-kB p65: *F*(1, 28) = 11.59, *p* = 0.0018; acetyl-NF-kB p65: *F*(1, 28) = 11.02, *p* = 0.0021; Sirt1: *F*(1, 28) = 10.47, *p* = 0.0027) compared to the MS group treated with saline.

**Fig. 6:**
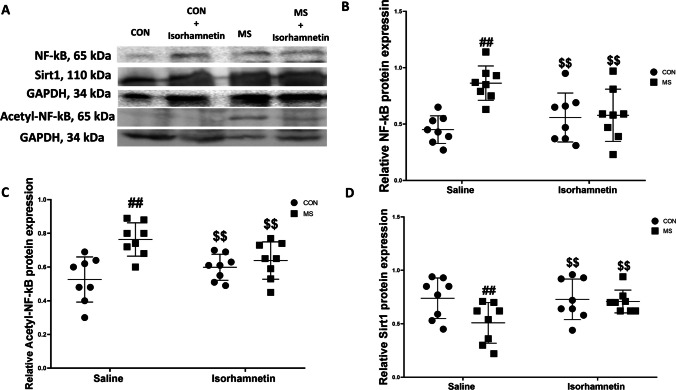
Isorhamnetin effectively prevented the maternal separation-induced decrease in Sirt1 protein levels and the concurrent increase in NF-kB p65 and acetyl-NF-kB p65 protein levels in the hippocampus. The assessment involved **A** Western blotting to measure the protein levels of NF-kB p65, acetyl-NF-kB p65, and Sirt1 in the hippocampus, and **B**–**D** protein quantification results, specifically for **B** NF-kB p65, **C** acetyl-NF-kB p65, and **D** Sirt1. All data are presented as means ± SEM. Significant comparisons are highlighted as follows: saline-treated MS group vs. saline-treated control group (##*p* < 0.01), isorhamnetin-treated control group vs. saline-treated control group (*p* > 0.05), and isorhamnetin-treated MS group vs. saline-treated MS group ($$*p* < 0.01). The study comprised eight subjects per group, with CON representing the control group and MS signifying maternal separation

Furthermore, there were no significant differences in the protein levels of Sirt1, NF-kB p65, and acetyl-NF-kB p65 between the control group treated with saline and the control group treated with isorhamnetin (NF-kB p65: *F*(1, 28) = 0.88, *p* = 0.354; acetyl-NF-kB p65: *F*(1, 28) = 0.92, *p* = 0.344; Sirt1: *F*(1, 28) = 0.95, *p* = 0.336). This indicates that isorhamnetin does not affect these proteins in the absence of stress. These findings, as illustrated in Fig. 6A–D, show that isorhamnetin treatment effectively mitigated the changes in protein expression triggered by MS, It brings the protein expression levels closer to those observed in the control group. This underscores isorhamnetin’s potential to regulate stress and inflammation-related protein expression pathways back to a baseline state.

## Discussion

The study shows that isorhamnetin effectively reduces depression-like and anxiety-like behaviours in male mice subjected to maternal separation. Moreover, isorhamnetin significantly decreases the elevated hippocampal levels of IL-1β, IL-6, and TNF-α and influences the expression of key genes and proteins related to neuroinflammation and stress responses. Specifically, it increases Sirt1 expression and reduces NF-kB p65 expression. These findings emphasize the potential of isorhamnetin as a treatment for stress-related psychiatric disorders by addressing both behavioral and molecular aspects of early-life stress.

 Stress in rodents in early life can result in behaviors similar to depression and anxiety in adults [41]. Our investigation revealed that offspring that experienced maternal separation exhibited noticeable behaviors resembling anxiety and depression, which were linked to increased levels of hippocampal cytokines and reduced activity of the Sirt1 and activation of NF-kB. Furthermore, the findings of this study suggest that isorhamnetin might alleviate the impacts of maternal separation by enhancing the Sirt1/NF-κB pathway. It is essential to recognize that contemporary societal factors can negatively influence the mother–child relationship, potentially leading to adverse outcomes in offspring due to disruptions during critical periods of brain development [42]. Using rodent models, researchers have found that maternal separation causes stress and alters brain activities, resulting in long-lasting changes in behavior [43–45]. The separation of mothers from their offspring for extended periods aggravates depressive behaviors in offspring at adult age in mice and triggers anxiety-related behaviors at adolescence in rats [46, 47]. The findings are backed by our research, which shows that adult male mice subjected to early-life mother’s separation display signs of depression and anxiety.

Nevertheless, it is essential to acknowledge the variability in existing research on how maternal separation affects adult anxiety and depression, with some suggesting no alterations or even decreases in these behaviors under specific circumstances [48, 49]. The disparities could be due to variations in the length and regularity of the periods of separation or differences in the types of mouse strains used. According to the findings of Huang et al. [26], our study involved subjecting the male offspring to a 4-h separation daily for 20 days, resulting in increased anxiety and depressive behaviors. Future research should focus on developing a standardized model for maternal separation that can reliably predict changes in behavior and consistently elucidate the underlying mechanisms.

Several studies have focused on unraveling the physiological processes linked to anxiety and depression, with a specific emphasis on the abnormalities in the monoamine transmission system [33–35]. Although standard treatments aim to boost monoamine function, they often carry side effects [50–52], indicating the need for alternatives. Isorhamnetin, a compound extracted from plants, exhibits the potential to address psychiatric disorders by targeting different neural pathways. Our study findings indicated that isorhamnetin possesses a distinct antidepressant-like effect, reversing stress-induced depression-like behavior and alleviating anxiety-like behaviors.

Inflammation is also implicated in anxiety and depression, with studies showing elevated levels of pro-inflammatory cytokines in individuals with depression [53]. Studies conducted with mouse models show that mice given TNF-α injections exhibit mood disorders [54, 55]. This connection links stress-induced neuroinflammation to impaired hippocampal neurogenesis and behavioral issues [56]. Our research aligns with these findings, indicating that off-springs being separated from the mother leads to heightened levels of pro-inflammatory cytokines, which are linked to anxiety and depression [57]. Sirt1, recognized for its protective roles, is correlated with anxiety and depression [58–60]. There are indications that Sirt1 activation can alleviate symptoms by reducing inflammatory pathways, such as NF-kB [61]. The NF-κB protein has a critical role in controlling inflammation and is also associated with various neuropsychiatric disorders [62]. Sirt1 can decrease the activity of NF-κB p65, which is necessary for NF-κB activation [63], thereby inhibiting the transcriptional effects of NF-κB [64]. Recent findings suggest that the activation of the Sirt1/NF-kB pathway by Polydatin, Sirt1 activator, can alleviate depression in mice by reducing inflammation in neurons [61].

Beyond the effects apparent in our study, isorhamnetin is known to undertake various molecular actions that could shed light on its behavioral and neurobiological effects. Aside from mediating the neuroinflammatory pathways, as well as the Sirt1/NF-κB pathway, Isorhamnetin was shown to have antioxidant properties that are useful in counterbalancing oxidative stress, a recurrent hallmark of neurodegenerative diseases and mood disorders [65, 66]. Studies have demonstrated that isorhamnetin not only increases levels of antioxidant enzymes, such as superoxide dismutase but also decreases levels of malondialdehyde, an indicator of lipid peroxidation [14, 67].

Moreover, isorhamnetin has been shown to modulate other molecular pathways crucial to neuronal health and function. For instance, it targets the PI3K/Akt pathway, which regulates cell survival and neuroplasticity. It has also been shown to inhibit the MAPK pathway, which is typically associated with inflammatory processes in the brain [68, 69]. These molecular interactions indeed indicate that the therapeutic potential of Isorhamnetin could contribute not only to treating stress-induced disorders but also to alleviating the symptoms of chronic neuroinflammation and even providing a neuroprotective effect in the setting of Alzheimer’s and other cognitive impairments.

For the assessment of depressive-like behaviors, we employed the Forced Swimming Test (FST) and the Tail Suspension Test (TST) in our mouse model. These tests are well known in preclinical studies to measure the time of immobility during exposure to antidepressant compounds, which has been classically interpreted as a marker of behavioral despair. Although the term “despair” is open to interpretation when applied to rodents [70], the immobility seen in such tests is believed to indicate a limited urge to escape following repeated failure that mirrors some human depressive symptomatology, such as withdrawal and a general sense of helplessness.

These tests have been chosen because they are well-characterized, widely used, and validated as practical tests to provide a preliminary assessment of antidepressant-like activity in compounds. However, we also recognize the limitations of ethology in these models. Immobility, too, maybe a conservation-withdrawal strategy, a natural adaptive response to inescapable stress, not pure despair. Our findings, nonetheless, are drawn within a neurobiological context that accounts for neuroinflammatory and cytokine level changes, thereby providing a more comprehensive perspective on the organism’s state beyond a behavioral window of despair.

To align with ethical research practices, we conducted these tests under stringent guidelines designed to minimize animal distress [71]. This included the implementation of humane endpoints, rigorous monitoring during testing, and ensuring that all personnel were trained in ethical animal care procedures. Despite these measures, we recognize the need for alternative methodologies that do not induce stress or potential harm to animal subjects.

To overcome the limitations of FTS and TST, future research will be needed to incorporate different behavioral assays that measure other dimensions of depression, including anhedonia or social interaction that gives a more complete picture of depressive-like states. Taking a multifaceted approach would enable further interpretation of the potential antidepressant properties of isorhamnetin as well as validate the notion that depression is a multi-dimensional disorder. As a result, effective treatment may be a combinative approach (and service) rather than a single agent therapy.

Given that the hippocampus is well characterized as a modulator of the stress response and has been shown to undergo neuroinflammatory alterations during early-life stress, our study focused primarily on the hippocampus [72]. The hippocampus, a critical area for memory and learning, is one of the brain regions most affected by neuroinflammatory processes and, therefore, a relevant brain region for interventions aimed at decreasing the display of stress-related behaviors [73].

Despite this, we are aware that the amygdala plays a crucial role in emotion processing and the modulation of fear and stress responses [74]. It is essential to note that the amygdala was not omitted due to oversight but rather because we intentionally excluded its analysis from the current work to enhance study focus and feasibility. This was also limited by the given available analyses and resources. Focusing on a single region enabled a more comprehensive interrogation of the hippocampal pathways than would be afforded by simultaneous interrogation of several other brain regions.

The present study emphasizes the effect of isorhamnetin on the Sirt1 and NF-κB signaling pathway networks involved in neuroimmune actions in early-life stress models. Notably, the stimulation of Sirt1 activity by isorhamnetin is significant. Sirt1 is a widely recognized neuroprotective factor whose effects are achieved through the deacetylation of relevant transcription factors and proteins critical for stress responses [75]. Isorhamnetin may activate Sirt1, leading to the deacetylation of the transcription factor NF-κB, a crucial component in inflammation. Under these conditions, the dual recruitment of downregulating factors directly interacts with the transcriptional activity of NF-κB and represses the expression of acute-phase cytokines, including TNF-α, IL-1β, and IL-6, in the hippocampus.

**Fig. 7:**
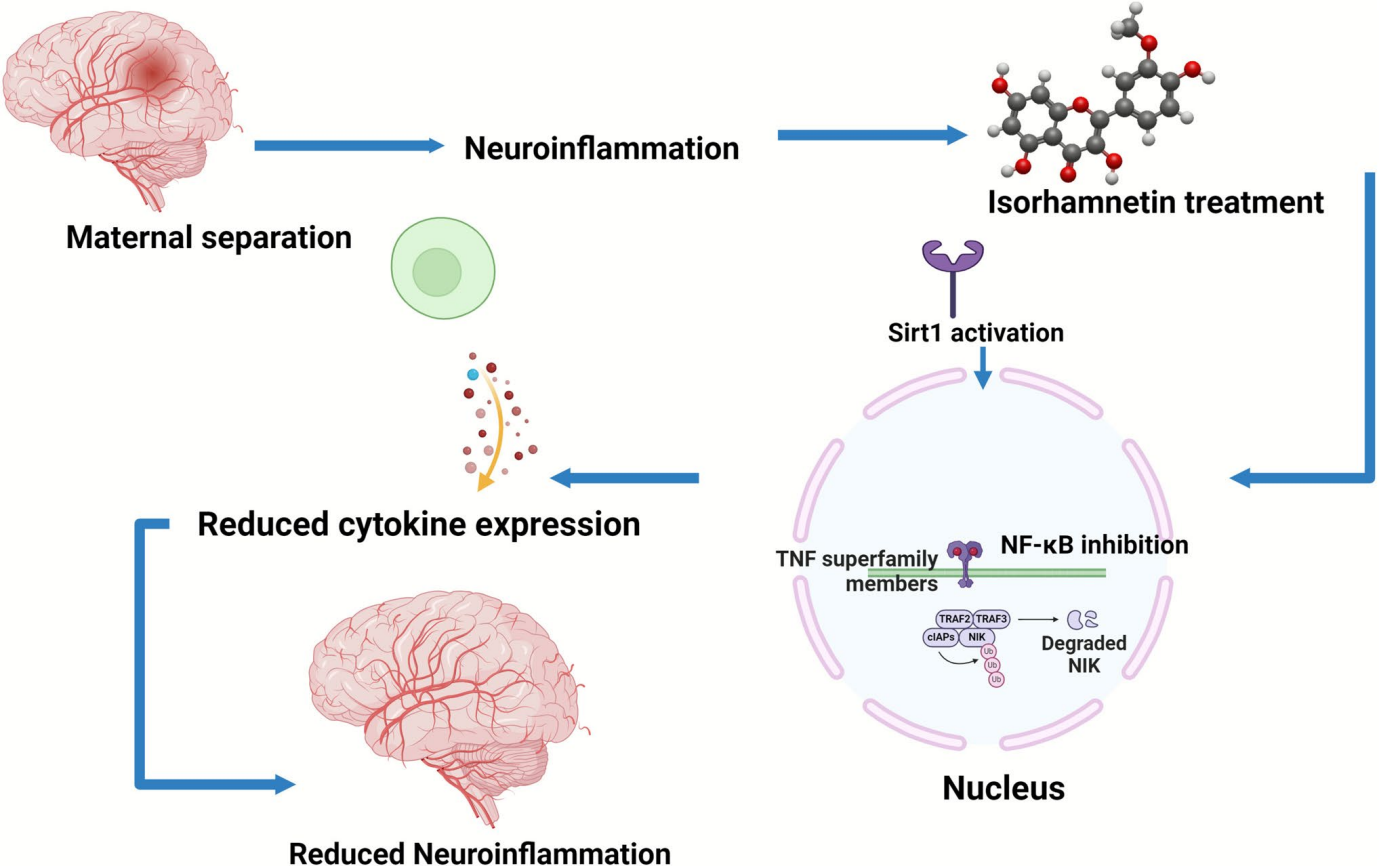
Schematic diagram illustrating how isorhamnetin modulates the Sirt1/NF-κB signaling pathway to alleviate neuroinflammation

Inhibition of NF-κB signaling by isorhamnetin is likely a critical mechanism by which the compound exerts its anti-inflammatory and potential antidepressant effects. Decreased inflammation in the hippocampus may help restore neuronal plasticity, which is crucial for both recovery and resilience to stress-related cognitive and emotional disorders [76]. Therefore, the enhancement in behavioral outcomes observed in our maternal separation model (reduced anxiety and depressive-like behaviors) may be attributed to the modulation of these pathways by isorhamnetin. Isorhamnetin treatment in the present study began at PND 61, which is beyond the time window of early-life stress (PND 2–21). This timing was intentionally chosen to model an intervention strategy that aims to revert, rather than prevent, the long-term consequences of early-life stress. From a translational point of view, this design more accurately reflects clinical reality in which the majority of psychiatric patients present with symptoms in adolescence or adulthood, many years after the stress exposure. These findings indicate that isorhamnetin remains effective when used after the establishment of both behavioral disturbances and the neuroinflammatory response. Hence, isorhamnetin may have therapeutic benefits in stress-related mood disorders. Our work, therefore, highlights the potential for isorhamnetin to be developed as an effective therapeutic strategy for treating neuroinflammatory and stress-related disorders. While it expands our understanding of the molecular substrates towards which psychiatric disorders may be targeted. Particularly in the case of isorhamnetin, the Sirt1 to NF-κB pathway are major mechanisms to manage early-life stress-induced psychiatric symptoms (Fig. 7).

In our investigation, we found that separation from the mother results in reduced Sirt1 elevated NF-kB p65 and acetylated NF-kB p65. However, the administration of isorhamnetin reversed these effects, indicating its potential to alleviate inflammation associated with separation through modulation of the Sirt1 and NF-κB signaling pathways. The limitations of our study include its exclusive focus on male offspring, which is prompted by concerns about how hormonal fluctuations in female mice may affect the outcomes of behavioral experiments [77, 78]. The exclusion of other brain regions, such as the amygdala, is also noticeable. To address these limitations, future research could encompass both sexes, explore additional brain regions. Furthermore, employing Sirt1 blockers or knockout models can be used to investigate the role of the Sirt1 and NF-kB signaling pathway in behavioral changes in maternally separated offspring and the effects of isorhamnetin under such conditions.

## Conclusion

Our research shows that early-life stress, such as being separated from the mother, has a significant impact on offspring to behaviors such as depression and anxiety at adult age. The hippocampus undergoes neuroinflammation as part of this process. Increased activity of the Sirt1 and NF-kB signaling pathway and the improvement in behavioral symptoms was observed with isorhamnetin treatment. Isorhamnetin has the potential as a new therapeutic approach for stress-related psychiatric conditions, potentially with fewer side effects than traditional treatments.

These findings underscore the need for further research to elucidate comprehensive underlying mechanisms. This can include female subjects and other brain regions associated with mood regulation. This will help to confirm the effectiveness of isorhamnetin as a treatment for stress-induced neuropsychiatric disorders and contribute to a comprehensive understanding of its therapeutic role.

## Supplementary Information

Below is the link to the electronic supplementary material.

ESM 1 (PDF 1.70 MB)

## Data Availability

The datasets generated during and/or analysed during the current study are available from the corresponding author upon reasonable request.

## References

[1] McEwen BS (2008) Understanding the potency of stressful early life experiences on brain and body function. Metabolism 57:S11–S15. 10.1016/J.METABOL.2008.07.006 18803958 10.1016/j.metabol.2008.07.006 PMC2567059

[2] Andersen SL (2015) Exposure to early adversity: points of cross-species translation that can lead to improved understanding of depression. Dev Psychopathol 27:477–491. 10.1017/S0954579415000103 25997766 10.1017/S0954579415000103 PMC5237807

[3] Hanson JL, Nacewicz BM, Sutterer MJ, Cayo AA, Schaefer SM, Rudolph KD et al (2015) Behavioral problems after early life stress: contributions of the hippocampus and amygdala. Biol Psychiatry 77:314–23. 10.1016/J.BIOPSYCH.2014.04.020 24993057 10.1016/j.biopsych.2014.04.020 PMC4241384

[4] Pillai AG, Arp M, Velzing E, Lesuis SL, Schmidt MV, Holsboer F et al (2018) Early life stress determines the effects of glucocorticoids and stress on hippocampal function: electrophysiological and behavioral evidence respectively. Neuropharmacology 133:307–318. 10.1016/J.NEUROPHARM.2018.02.001 29412144 10.1016/j.neuropharm.2018.02.001

[5] Fang H, Li J, Lu L, Yang J, Feng H, Yin X et al (2023) Long-lasting and sex-dependent effects of late lactational maternal deprivation on socioemotional behaviors in adult mice. Neurosci Lett 799:137096. 10.1016/J.NEULET.2023.137096 36738955 10.1016/j.neulet.2023.137096

[6] Juruena MF, Eror F, Cleare AJ, Young AH (2020) The role of early life stress in HPA axis and anxiety. Adv Exp Med Biol 1191:141–153. 10.1007/978-981-32-9705-0_9 32002927 10.1007/978-981-32-9705-0_9

[7] Zheng ZH, Tu JL, Li XH, Hua Q, Liu WZ, Liu Y et al (2021) Neuroinflammation induces anxiety- and depressive-like behavior by modulating neuronal plasticity in the basolateral amygdala. Brain Behav Immun 91:505–18. 10.1016/J.BBI.2020.11.007 33161163 10.1016/j.bbi.2020.11.007

[8] Andersen SL (2022) Neuroinflammation, early-life adversity, and brain development. Harv Rev Psychiatry 30:24–39. 10.1097/HRP.0000000000000325 34995033 10.1097/HRP.0000000000000325 PMC8820591

[9] Pandey GN (2017) Inflammatory and innate immune markers of neuroprogression in depressed and teenage suicide brain. Mod Trends Pharmacopsychiatry 31:79–95. 10.1159/000470809 28738369 10.1159/000470809

[10] Mi X, Zeng GR, Liu JQ, Luo ZS, Zhang L, Dai XM et al (2022) *Ganoderma lucidum* triterpenoids improve maternal separation-induced anxiety-and depression-like behaviors in mice by mitigating inflammation in the periphery and brain. Nutrients 14:2268. 10.3390/NU14112268/S1 35684068 10.3390/nu14112268 PMC9182879

[11] Arabi M, Nasab SH, Lorigooini Z, Boroujeni SN, Mortazavi SM, Anjomshoa M et al (2021) Auraptene exerts protective effects on maternal separation stress-induced changes in behavior, hippocampus, heart and serum of mice. Int Immunopharmacol 93:107436. 10.1016/J.INTIMP.2021.107436 33556667 10.1016/j.intimp.2021.107436

[12] Rostami-Faradonbeh N, Amini-Khoei H, Zarean E, Bijad E, Lorigooini Z (2024) Anethole as a promising antidepressant for maternal separation stress in mice by modulating oxidative stress and nitrite imbalance. Sci Rep 14(1):1–10. 10.1038/s41598-024-57959-2 38565927 10.1038/s41598-024-57959-2 PMC10987547

[13] Nouri A, Hashemzadeh F, Soltani A, Saghaei E, Amini-Khoei H (2020) Progesterone exerts antidepressant-like effect in a mouse model of maternal separation stress through mitigation of neuroinflammatory response and oxidative stress. Pharm Biol 58:64–71. 10.1080/13880209.2019.1702704 31873049 10.1080/13880209.2019.1702704 PMC6968520

[14] Alqudah A, Qnais EY, Wedyan MA, Altaber S, Bseiso Y, Oqal M et al (2023) Isorhamnetin reduces glucose level, inflammation, and oxidative stress in high-fat diet/streptozotocin diabetic mice model. Molecules 28(2):502. 10.3390/MOLECULES28020502 36677559 10.3390/molecules28020502 PMC9866402

[15] Li C, Yang X, Chen C, Cai S, Hu J (2014) Isorhamnetin suppresses colon cancer cell growth through the PI3K-Akt-mTOR pathway. Mol Med Rep 9:935–940. 10.3892/MMR.2014.1886/HTML 24398569 10.3892/mmr.2014.1886

[16] Yokozawa T, Kim HY, Cho EJ, Choi JS, Chung HY (2002) Antioxidant effects of isorhamnetin 3,7-di-O-β-d-glucopyranoside isolated from mustard leaf (*Brassica juncea*) in rats with streptozotocin-induced diabetes. J Agric Food Chem 50:5490–5. 10.1021/JF0202133 12207497 10.1021/jf0202133

[17] Jamali-Raeufy N, Baluchnejadmojarad T, Roghani M, keimasi S, goudarzi M (2019) Isorhamnetin exerts neuroprotective effects in STZ-induced diabetic rats via attenuation of oxidative stress, inflammation and apoptosis. J Chem Neuroanat 102:101709. 10.1016/J.JCHEMNEU.2019.101709 31698018 10.1016/j.jchemneu.2019.101709

[18] Wei PC, Lee-Chen GJ, Chen CM, Chen Y, Lo YS, Chang KH (2022) Isorhamnetin attenuated the release of interleukin-6 from β -amyloid-activated microglia and mitigated interleukin-6-mediated neurotoxicity. Oxid Med Cell Longev. 10.1155/2022/3652402 36160711 10.1155/2022/3652402 PMC9499806

[19] Xu SL, Choi RCY, Zhu KY, Leung KW, Guo AJY, Bi D et al (2012) Isorhamnetin, a flavonol aglycone from *Ginkgo biloba* L., induces neuronal differentiation of cultured PC12 cells: potentiating the effect of nerve growth factor. Evid Based Complement Alternat Med. 10.1155/2012/278273 22761636 10.1155/2012/278273 PMC3385709

[20] Gammoh O, Qnais EY, Athamneh RY, Al-Jaidi B, Al-Tawalbeh D, Altaber S et al (2023) Unraveling the potential of isorhamnetin as an adjuvant in depression treatment with escitalopram. Curr Issues Mol Biol 45(9):7668–7679. 10.3390/CIMB45090484 37754268 10.3390/cimb45090484 PMC10530211

[21] Wang J, Gong HM, Zou HH, Liang L, Wu XY (2018) Isorhamnetin prevents H2O2-induced oxidative stress in human retinal pigment epithelial cells. Mol Med Rep 17:648–52. 10.3892/mmr.2017.7916 29115489 10.3892/mmr.2017.7916

[22] Mulati A, Zhang X, Zhao T, Ren B, Wang L, Liu X et al (2021) Isorhamnetin attenuates high-fat and high-fructose diet induced cognitive impairments and neuroinflammation by mediating MAPK and NFκB signaling pathways. Food Funct 12:9261–9272. 10.1039/D0FO03165H 34606526 10.1039/d0fo03165h

[23] Wu Y, Fan L, Wang Y, Ding J, Wang R (2021) Isorhamnetin alleviates high glucose-aggravated inflammatory response and apoptosis in oxygen-glucose deprivation and reoxygenation-induced HT22 hippocampal neurons through Akt/SIRT1/Nrf2/HO-1 signaling pathway. Inflammation 44(5):1993–2005. 10.1007/S10753-021-01476-1 33999329 10.1007/s10753-021-01476-1

[24] Deng X, Ren T, Zhang H, Gao S, Yang W, Zhang J, Yu H, Jin X, et al. (2023) Isorhamnetin alleviates the depression induced by hindlimb unloading in rats. Microgravity Sci Technol 35(5):1–12. 10.1007/S12217-023-10070-Z

[25] Ekici M, Gungor H, Mert DG (2023) Kaempferol and isorhamnetin alleviate lipopolysaccharide-induced anxiety and depression-like behavioral in Balb/C mice. J Hellenic Vet Med Soc 74:5749–5760. 10.12681/jhvms.30232

[26] Huang H, Wang Q, Guan X, Zhang X, Zhang Y, Cao J et al (2021) Effects of enriched environment on depression and anxiety-like behavior induced by early life stress: a comparison between different periods. Behav Brain Res 411:113389. 10.1016/J.BBR.2021.113389 34058267 10.1016/j.bbr.2021.113389

[27] Wei RM, Zhang YM, Li Y, Wu QT, Wang YT, Li XY, Li X-W, Chen G-H (2022) Altered cognition and anxiety in adolescent offspring whose mothers underwent different-pattern maternal sleep deprivation, and cognition link to hippocampal expressions of Bdnf and Syt-1. Front Behav Neurosci 16:1066725. 10.3389/FNBEH.2022.1066725 36570704 10.3389/fnbeh.2022.1066725 PMC9772274

[28] Alqudah A, Qnais E, Gammoh O, Bseiso Y, Wedyan M, Alqudah M, et al (2024) Scopoletin mitigates maternal separation-induced anxiety-like and depression-like behaviors in male mice through modulation of the Sirt1/NF-κB pathway. Psychopharmacol (Berl) 1–16. 10.1007/S00213-024-06639-0/FIGURES/1 10.1007/s00213-024-06639-0 38886190

[29] Wei RM, Zhang YM, Feng YZ, Zhang KX, Zhang JY, Chen J et al (2023) Resveratrol ameliorates maternal separation-induced anxiety- and depression-like behaviors and reduces Sirt1-NF-kB signaling-mediated neuroinflammation. Front Behav Neurosci 17:1–11. 10.3389/fnbeh.2023.1172091 10.3389/fnbeh.2023.1172091 PMC10233157 37273278

[30] Ueno H, Takahashi Y, Murakami S, Wani K, Matsumoto Y, Okamoto M et al (2022) Effect of simultaneous testing of two mice in the tail suspension test and forced swim test. Sci Rep 12(1):1–14. 10.1038/s41598-022-12986-9 35654971 10.1038/s41598-022-12986-9 PMC9163059

[31] Castagné V, Moser P, Roux S, Porsolt RD (2010) Rodent models of depression: forced swim and tail suspension behavioral despair tests in rats and mice. Curr Protoc Pharmacol 49:5.8.1-5.8.14. 10.1002/0471141755.PH0508S49 10.1002/0471141755.ph0508s49 22294373

[32] Walf AA, Frye CA (2007) The use of the elevated plus maze as an assay of anxiety-related behavior in rodents. Nat Protoc 2(2):322–328. 10.1038/NPROT.2007.44 17406592 10.1038/nprot.2007.44 PMC3623971

[33] Laferriere CA, Pang DS (2020) Review of intraperitoneal injection of sodium pentobarbital as a method of euthanasia in laboratory rodents. J Am Assoc Lab Anim Sci 59:254. 10.30802/AALAS-JAALAS-19-000081 10.30802/AALAS-JAALAS-19-000081 PMC7210732 32156325

[34] Matsubara NK, Silva-Santos JEda (2024) The dual cardiovascular effect of centrally administered clonidine: a comparative study between pentobarbital- and ketamine/xylazine-anesthetized rats. Futur Pharmacol 4:17–29. 10.3390/FUTUREPHARMACOL4010003

[35] Yang FL, Li CH, Hsu BG, Tsai NM, Lin SZ, Harn HJ et al (2007) The reduction of tumor necrosis factor-α release and tissue damage by pentobarbital in the experimental endotoxemia model. Shock 28:309–16. 10.1097/SHK.0B013E31803DD04D 17545946 10.1097/SHK.0b013e31803dd04d

[36] Onizuka C, Irifune M, Mukai A, Shimizu Y, Doi M, Oue K et al (2022) Pentobarbital may protect against neurogenic inflammation after surgery via inhibition of substance P release from peripheral nerves of rats. Neurosci Lett 771:136467. 10.1016/J.NEULET.2022.136467 35063502 10.1016/j.neulet.2022.136467

[37] Subeq YM, Wu WT, Lee CJ, Lee RP, Yang FL, Hsu BG (2009) Pentobarbital reduces rhabdomyolysis-induced acute renal failure in conscious rats. J Trauma Inj 67:132–138. 10.1097/TA.0B013E318186253D 10.1097/TA.0b013e318186253d 19590322

[38] Zhang YM, Cheng YZ, Wang YT, Wei RM, Ge YJ, Kong XY, Li X-Y (2022) Environmental enrichment reverses maternal sleep deprivation-induced anxiety-like behavior and cognitive impairment in CD-1 mice. Front Behav Neurosci 16:943900. 10.3389/FNBEH.2022.943900 35910680 10.3389/fnbeh.2022.943900 PMC9326347

[39] Calvo AC, Moreno-Igoa M, Manzano R, Ordovás L, Yagüe G, Oliván S et al (2008) Determination of protein and RNA expression levels of common housekeeping genes in a mouse model of neurodegeneration. Proteomics 8:4338–43. 10.1002/PMIC.200701091 18814324 10.1002/pmic.200701091

[40] Livak KJ, Schmittgen TD (2001) Analysis of relative gene expression data using real-time quantitative PCR and the 2−ΔΔCT method. Methods 25:402–408. 10.1006/METH.2001.1262 11846609 10.1006/meth.2001.1262

[41] Godoy LD, Umeoka EHL, Ribeiro DE, Santos VR, Antunes-Rodrigues J, Joca SRL et al (2018) Multimodal early-life stress induces biological changes associated to psychopathologies. Horm Behav 100:69–80. 10.1016/J.YHBEH.2018.03.005 29548783 10.1016/j.yhbeh.2018.03.005

[42] Bergman NJ (2019) Birth practices: maternal-neonate separation as a source of toxic stress. Birth Defects Res 111:1087–1109. 10.1002/BDR2.1530 31157520 10.1002/bdr2.1530

[43] Nishi M, Horii-Hayashi N, Sasagawa T (2014) Effects of early life adverse experiences on the brain: implications from maternal separation models in rodents. Front Neurosci 8:87697. 10.3389/FNINS.2014.00166/BIBTEX 10.3389/fnins.2014.00166 PMC4060417 24987328

[44] Zhang B, Wang H, Yang Z, Cao M, Wang K, Wang G et al (2020) Protective effect of alpha-pinene against isoproterenol-induced myocardial infarction through NF-κB signaling pathway. Hum Exp Toxicol 39:1596–1606. 10.1177/0960327120934537/ASSET/IMAGES/LARGE/10.1177_0960327120934537-FIG4.JPEG 32602371 10.1177/0960327120934537

[45] Tsotsokou G, Nikolakopoulou M, Kouvelas ED, Mitsacos A (2021) Neonatal maternal separation affects metabotropic glutamate receptor 5 expression and anxiety-related behavior of adult rats. Eur J Neurosci 54:4550–4564. 10.1111/EJN.15358 34137089 10.1111/ejn.15358

[46] Zhou L, Wu Z, Wang G, Xiao L, Wang H, Sun L et al (2020) Long-term maternal separation potentiates depressive-like behaviours and neuroinflammation in adult male C57/BL6J mice. Pharmacol Biochem Behav 196:172953. 10.1016/J.PBB.2020.172953 32450088 10.1016/j.pbb.2020.172953

[47] Zhang N, Zhang R, Loers G, Liu C, Jin L, Petridis AK et al (2020) Cuprizone-induced demyelination in mouse hippocampus is alleviated by ketogenic diet. J Agric Food Chem 68:11215–28. 10.1021/ACS.JAFC.0C04604 32921051 10.1021/acs.jafc.0c04604

[48] Zhou L, Wu Z, Li Y, Xiao L, Wang H, Wang G (2022) Brief maternal separation promotes resilience to anxiety-like and depressive-like behaviors in female C57BL/6J offspring with imiquimod-induced psoriasis. Brain Sci 12:1250. 10.3390/BRAINSCI12091250 36138986 10.3390/brainsci12091250 PMC9497052

[49] Jarrar Q, Ayoub R, Alhussine K, Goh KW, Moshawih S, Ardianto C et al (2022) Prolonged maternal separation reduces anxiety state and increases compulsive burying activity in the offspring of BALB/c mice. J Pers Med 12:1921. 10.3390/JPM12111921 36422097 10.3390/jpm12111921 PMC9699014

[50] Kenda M, Kočevar Glavač N, Nagy M, Sollner Dolenc M (2022) Medicinal plants used for anxiety, depression, or stress treatment: an update. Molecules 27:6021. 10.3390/MOLECULES27186021 36144755 10.3390/molecules27186021 PMC9500625

[51] Stahl SM (1998) Mechanism of action of serotonin selective reuptake inhibitors: serotonin receptors and pathways mediate therapeutic effects and side effects. J Affect Disord 51:215–235. 10.1016/S0165-0327(98)00221-3 10333979 10.1016/s0165-0327(98)00221-3

[52] Yeung KS, Hernandez M, Mao JJ, Haviland I, Gubili J (2018) Herbal medicine for depression and anxiety: a systematic review with assessment of potential psycho-oncologic relevance. Phyther Res 32:865–891. 10.1002/PTR.6033 10.1002/ptr.6033 PMC5938102 29464801

[53] Petralia MC, Mazzon E, Fagone P, Basile MS, Lenzo V, Quattropani MC et al (2020) The cytokine network in the pathogenesis of major depressive disorder. Close to translation? Autoimmun Rev 19:102504. 10.1016/J.AUTREV.2020.102504 32173514 10.1016/j.autrev.2020.102504

[54] Ma K, Zhang H, Baloch Z (2016) Pathogenetic and therapeutic applications of tumor necrosis factor-α (TNF-α) in major depressive disorder: a systematic review. Int J Mol Sci 17:733. 10.3390/IJMS17050733 27187381 10.3390/ijms17050733 PMC4881555

[55] Haji N, Mandolesi G, Gentile A, Sacchetti L, Fresegna D, Rossi S et al (2012) TNF-α-mediated anxiety in a mouse model of multiple sclerosis. Exp Neurol 237:296–303. 10.1016/J.EXPNEUROL.2012.07.010 22836148 10.1016/j.expneurol.2012.07.010

[56] McKim DB, Niraula A, Tarr AJ, Wohleb ES, Sheridan JF, Godbout JP (2016) Neuroinflammatory dynamics underlie memory impairments after repeated social defeat. J Neurosci 36:2590–2604. 10.1523/JNEUROSCI.2394-15.2016 26937001 10.1523/JNEUROSCI.2394-15.2016 PMC4879207

[57] Wang R, Wang W, Xu J, Liu D, Wu H, Qin X et al (2020) Jmjd3 is involved in the susceptibility to depression induced by maternal separation via enhancing the neuroinflammation in the prefrontal cortex and hippocampus of male rats. Exp Neurol 328:113254. 10.1016/J.EXPNEUROL.2020.113254 32084453 10.1016/j.expneurol.2020.113254

[58] Sakr HF, Abbas AM, Elsamanoudy AZ, Ghoneim FM (2015) Effect of fluoxetine and resveratrol on testicular functions and oxidative stress in a rat model of chronic mild stress-induced depression. J Physiol Pharmacol 66:515–527 26348076

[59] Abe-Higuchi N, Uchida S, Yamagata H, Higuchi F, Hobara T, Hara K et al (2016) Hippocampal sirtuin 1 signaling mediates depression-like behavior. Biol Psychiatry 80:815–26. 10.1016/J.BIOPSYCH.2016.01.009 27016384 10.1016/j.biopsych.2016.01.009

[60] Yu D, Homiack DR, Sawyer EJ, Schrader LA (2018) BK channel deacetylation by SIRT1 in dentate gyrus regulates anxiety and response to stress. Commun Biol 1:1–14. 10.1038/s42003-018-0088-5 30271963 10.1038/s42003-018-0088-5 PMC6123630

[61] Bian H, Xiao L, Liang L, Xie Y, Wang H, Slevin M et al (2022) Polydatin prevents neuroinflammation and relieves depression via regulating Sirt1/HMGB1/NF-κB signaling in mice. Neurotox Res 40:1393–1404. 10.1007/S12640-022-00553-Z/FIGURES/8 35986876 10.1007/s12640-022-00553-z

[62] Yang J, Liu R, Lu F, Xu F, Zheng J, Li Z et al (2019) Fast green FCF attenuates lipopolysaccharide-induced depressive-like behavior and downregulates TLR4/Myd88/NF-κB signal pathway in the mouse hippocampus. Front Pharmacol 10:445064. 10.3389/FPHAR.2019.00501/BIBTEX 10.3389/fphar.2019.00501 PMC6519320 31139084

[63] Kauppinen A, Suuronen T, Ojala J, Kaarniranta K, Salminen A (2013) Antagonistic crosstalk between NF-κB and SIRT1 in the regulation of inflammation and metabolic disorders. Cell Signal 25(10):1939–1948. 10.1016/J.CELLSIG.2013.06.007 23770291 10.1016/j.cellsig.2013.06.007

[64] Yeung F, Hoberg JE, Ramsey CS, Keller MD, Jones DR, Frye RA et al (2004) Modulation of NF-κB-dependent transcription and cell survival by the SIRT1 deacetylase. EMBO J 23:2369–2380. 10.1038/SJ.EMBOJ.7600244/ASSET/D1ECDA08-8839-406E-8C1D-16A7AAF687EA/ASSETS/GRAPHIC/EMBJ7600244-FIG-0008-M.JPG 15152190 10.1038/sj.emboj.7600244 PMC423286

[65] Wadhwa R, Gupta R, Maurya PK (2019) Oxidative stress and accelerated aging in neurodegenerative and neuropsychiatric disorder. Curr Pharm Des 24:4711–4725. 10.2174/1381612825666190115121018 10.2174/1381612825666190115121018 30644343

[66] Yang JH, Shin BY, Han JY, Kim MG, Wi JE, Kim YW et al (2014) Isorhamnetin protects against oxidative stress by activating Nrf2 and inducing the expression of its target genes. Toxicol Appl Pharmacol 274:293–301. 10.1016/J.TAAP.2013.10.026 24211276 10.1016/j.taap.2013.10.026

[67] Zhao TT, Yang TL, Gong L, Wu P (2018) Isorhamnetin protects against hypoxia/reoxygenation-induced injure by attenuating apoptosis and oxidative stress in H9c2 cardiomyocytes. Gene 666:92–99. 10.1016/J.GENE.2018.05.009 29730426 10.1016/j.gene.2018.05.009

[68] Gao L, Yao R, Liu Y, Wang Z, Huang Z, Du B et al (2017) Isorhamnetin protects against cardiac hypertrophy through blocking PI3K–AKT pathway. Mol Cell Biochem 429:167–177. 10.1007/S11010-017-2944-X/FIGURES/6 28176246 10.1007/s11010-017-2944-x

[69] Gong G, Guan YY, Zhang ZL, Rahman K, Wang SJ, Zhou S et al (2020) Isorhamnetin: a review of pharmacological effects. Biomed Pharmacother 128:110301. 10.1016/J.BIOPHA.2020.110301 32502837 10.1016/j.biopha.2020.110301

[70] Commons KG, Cholanians AB, Babb JA, Ehlinger DG (2017) The rodent forced swim test measures stress-coping strategy, not depression-like behavior. ACS Chem Neurosci 8:955–60. 10.1021/ACSCHEMNEURO.7B00042/ASSET/IMAGES/MEDIUM/CN-2017-00042V_0003.GIF 28287253 10.1021/acschemneuro.7b00042 PMC5518600

[71] Animal Research Amendment (Prohibition of Forced Swim Tests & Forced Smoke Inhalation Experiments) 2024 (n.d.) https://www.dpi.nsw.gov.au/animals-and-livestock/animal-welfare/animal-welfare-reform/AR-amendment-forced-swim-smoke-tests.. Accessed 9 Jan 2025

[72] Wingenfeld K, Wolf OT (2014) Stress, memory, and the hippocampus. Hippocampus Clin Neurosci 34:109–120. 10.1159/000356423 10.1159/000356423 24777135

[73] Miller DB, O’Callaghan JP (2005) Aging, stress and the hippocampus. Ageing Res Rev 4:123–140. 10.1016/J.ARR.2005.03.002 15964248 10.1016/j.arr.2005.03.002

[74] Roozendaal B, McEwen BS, Chattarji S (2009) Stress, memory and the amygdala. Nat Rev Neurosci 10(6):423–433. 10.1038/nrn2651 19469026 10.1038/nrn2651

[75] Nimmagadda VKC, Makar TK, Chandrasekaran K, Sagi AR, Ray J, Russell JW et al (2017) SIRT1 and NAD+ precursors: therapeutic targets in multiple sclerosis a review. J Neuroimmunol 304:29–34. 10.1016/J.JNEUROIM.2016.07.007 27474445 10.1016/j.jneuroim.2016.07.007 PMC5528149

[76] Cattaneo A, Macchi F, Plazzotta G, Veronica B, Bocchio-Chiavetto L, Riva MA et al (2015) Inflammation and neuronal plasticity: a link between childhood trauma and depression pathogenesis. Front Cell Neurosci 9:113480. 10.3389/FNCEL.2015.00040/BIBTEX 10.3389/fncel.2015.00040 PMC4379909 25873859

[77] Wei Y, Wang G, Wang H, He J, Zhang N, Wu Z et al (2018) Sex-dependent impact of different degrees of maternal separation experience on OFT behavioral performances after adult chronic unpredictable mild stress exposure in rats. Physiol Behav 194:153–61. 10.1016/J.PHYSBEH.2018.04.034 29723593 10.1016/j.physbeh.2018.04.034

[78] Kim R, Islam MS, Yoo YJ, Shin HY, Lee JH, Cho JH et al (2022) Anti-inflammatory effects of the Aralia elata and Cirsium japonicum in Raw264.7 cells and in vivo colitis model in mice and dogs. Biomed Pharmacother 151:113186. 10.1016/J.BIOPHA.2022.113186 35643063 10.1016/j.biopha.2022.113186

