# Supplementary figures and images for: Isorhamnetin Alleviates Early-Life Stress-Induced Anxiety and Depression in Male Mice: Neuroinflammatory Modulation and Sirt1/NF-κB Signaling Insights
Source: Mol Neurobiol. 2025 Nov 29;63(1):216. doi: 10.1007/s12035-025-05483-3 (PMC12664856; doi:10.1007/s12035-025-05483-3)

**Sirt1, 110 kDa**

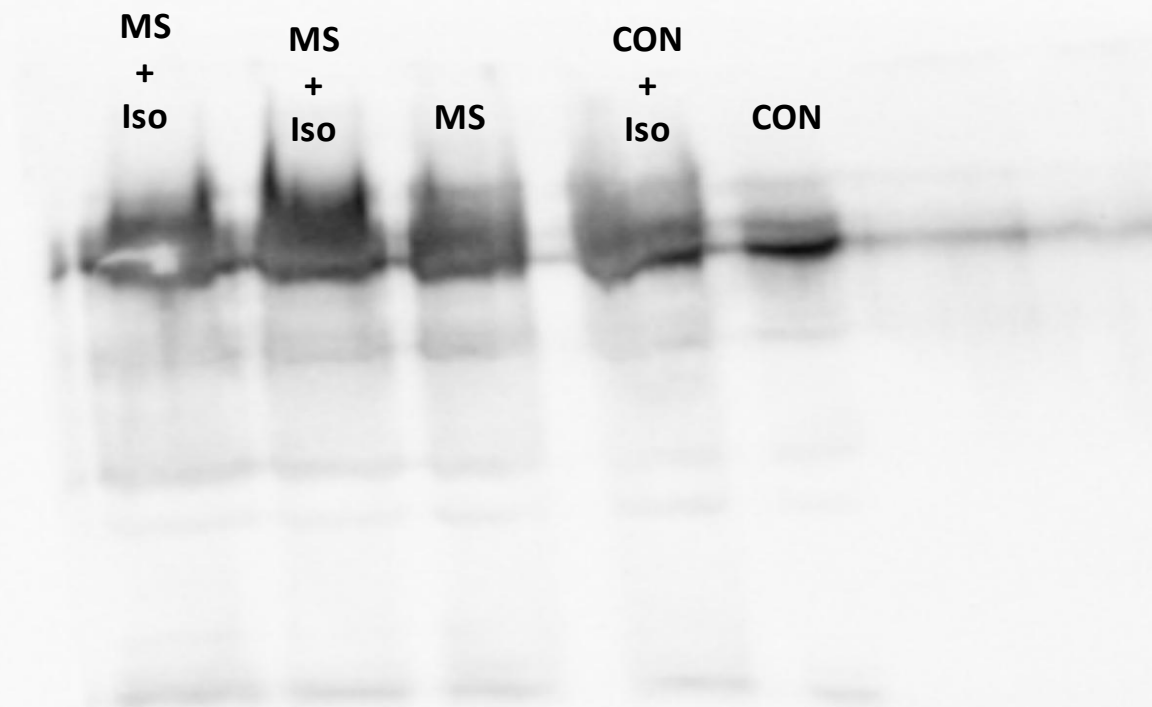

MS  
+  
Iso

MS  
+  
Iso

MS

CON  
+  
Iso

CON

NF-kB, 65 kDa

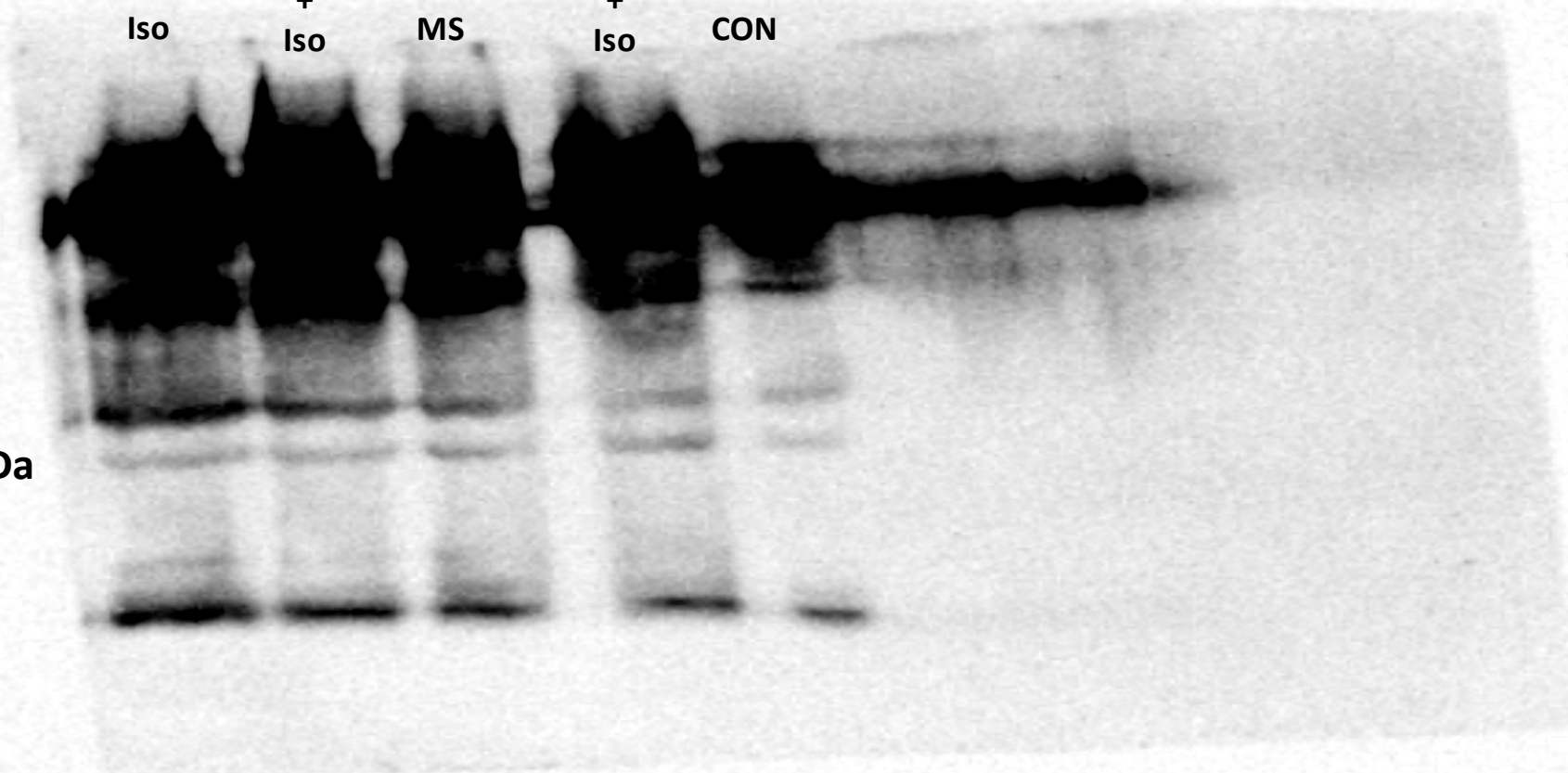

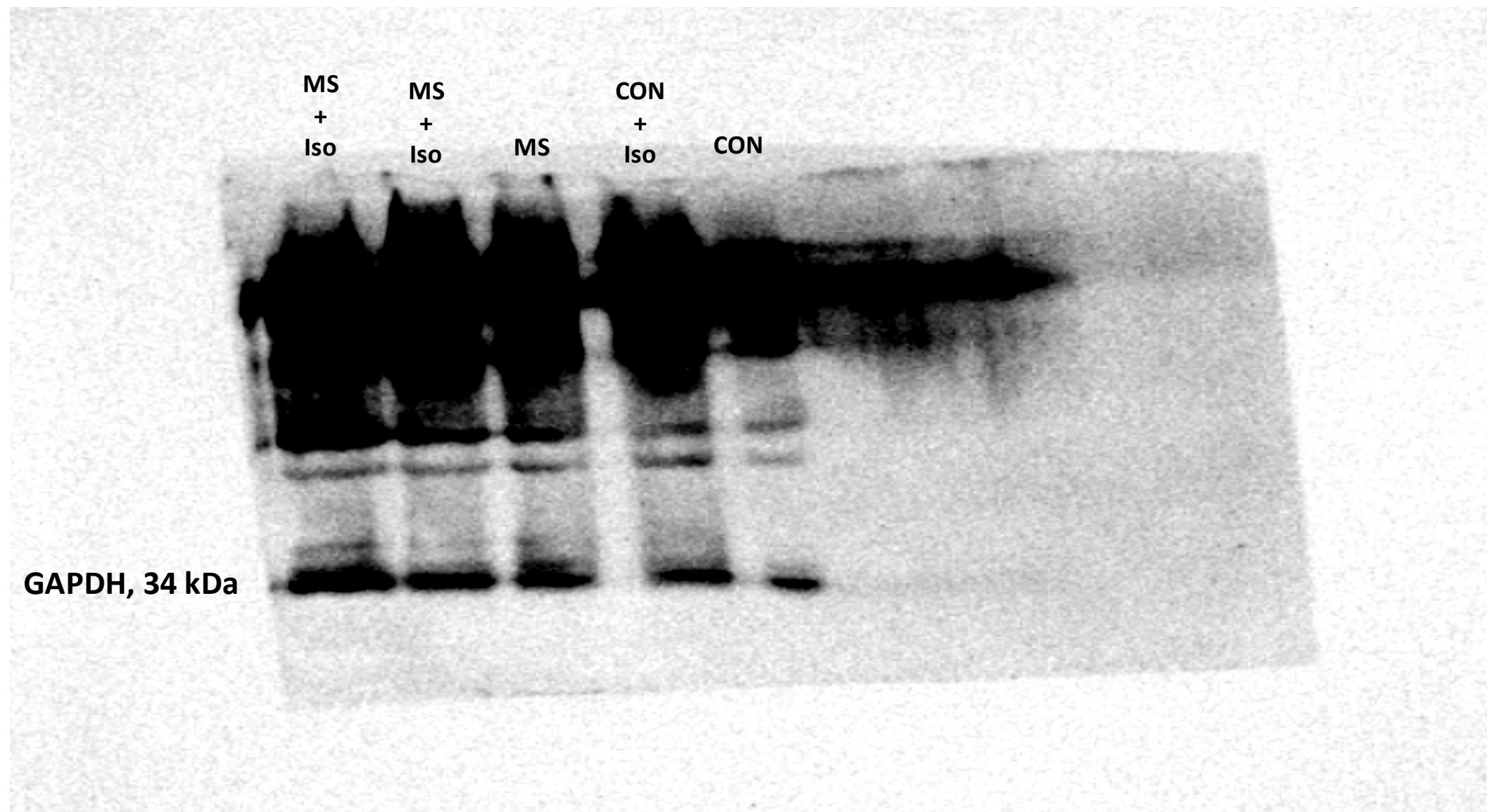

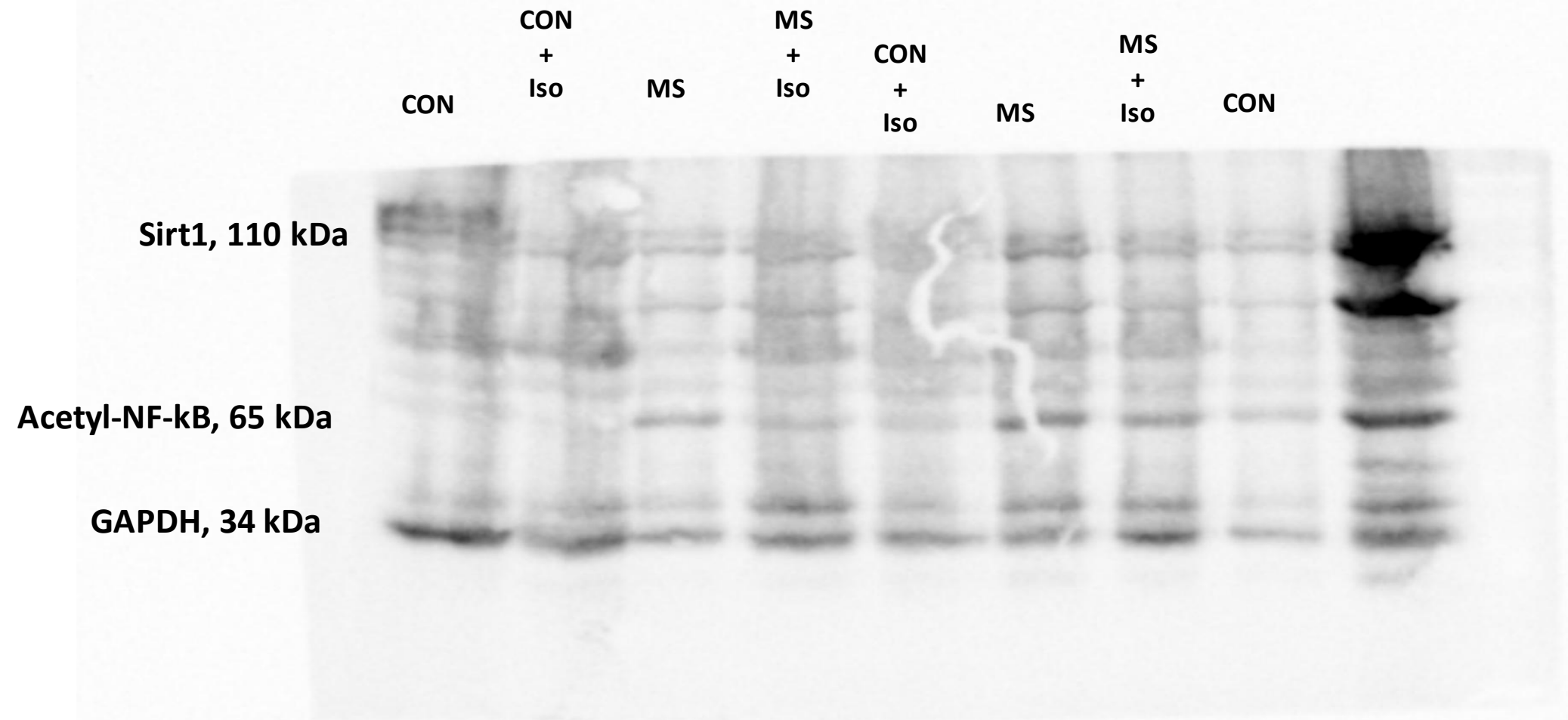

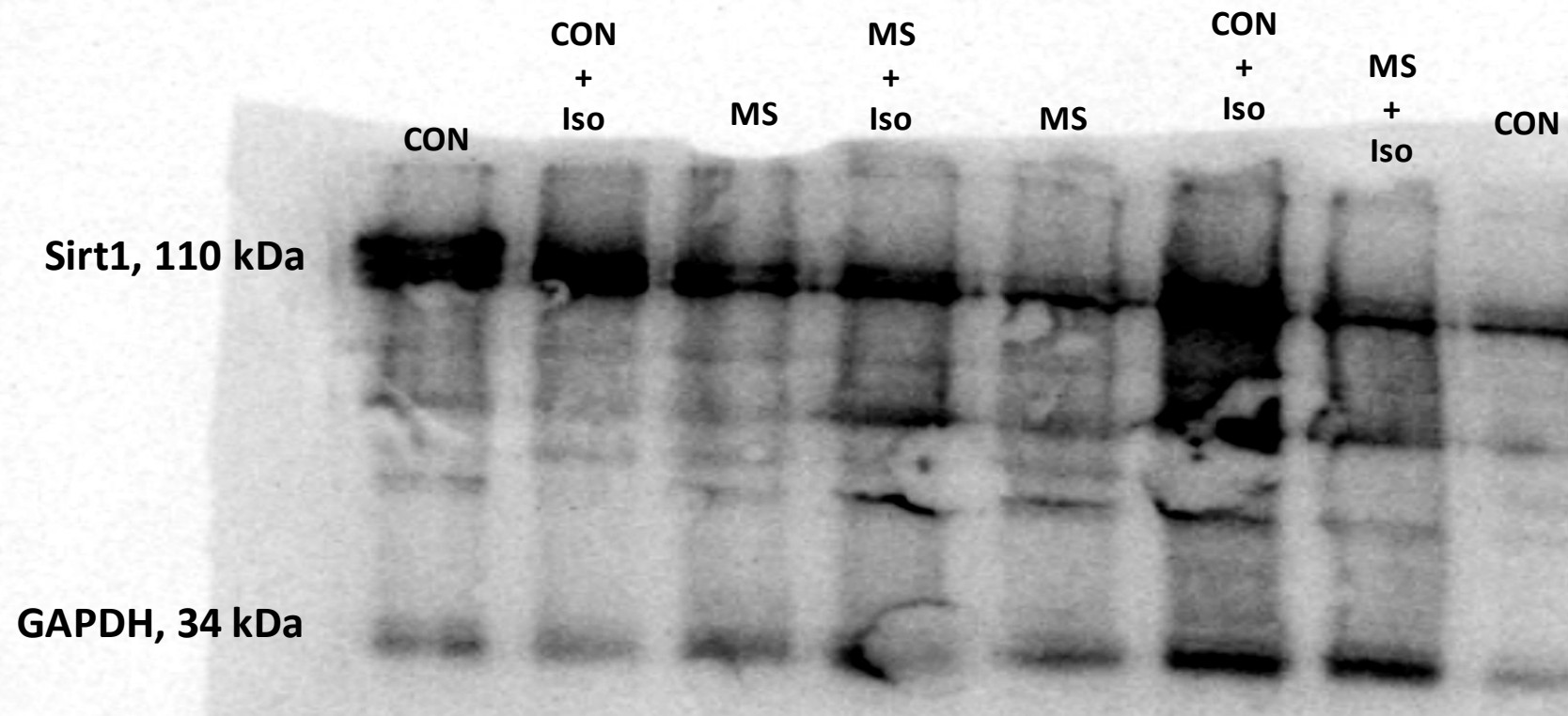



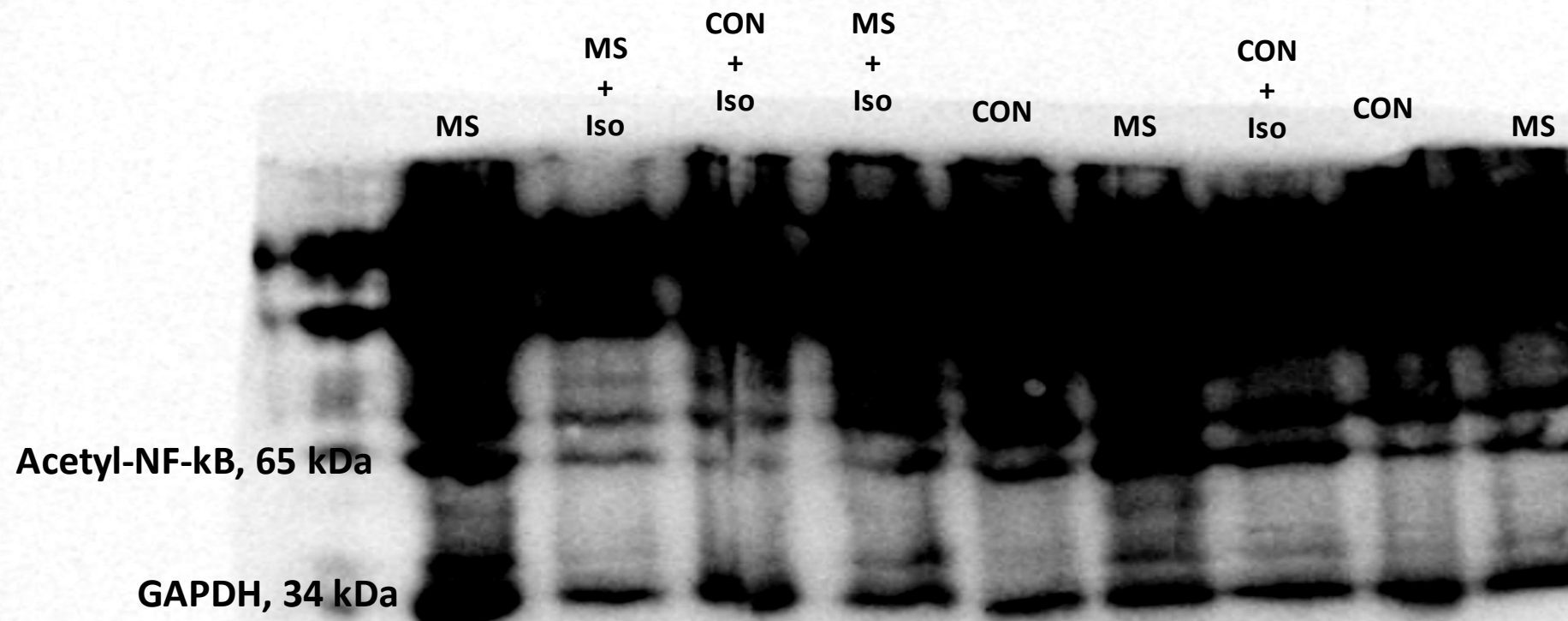



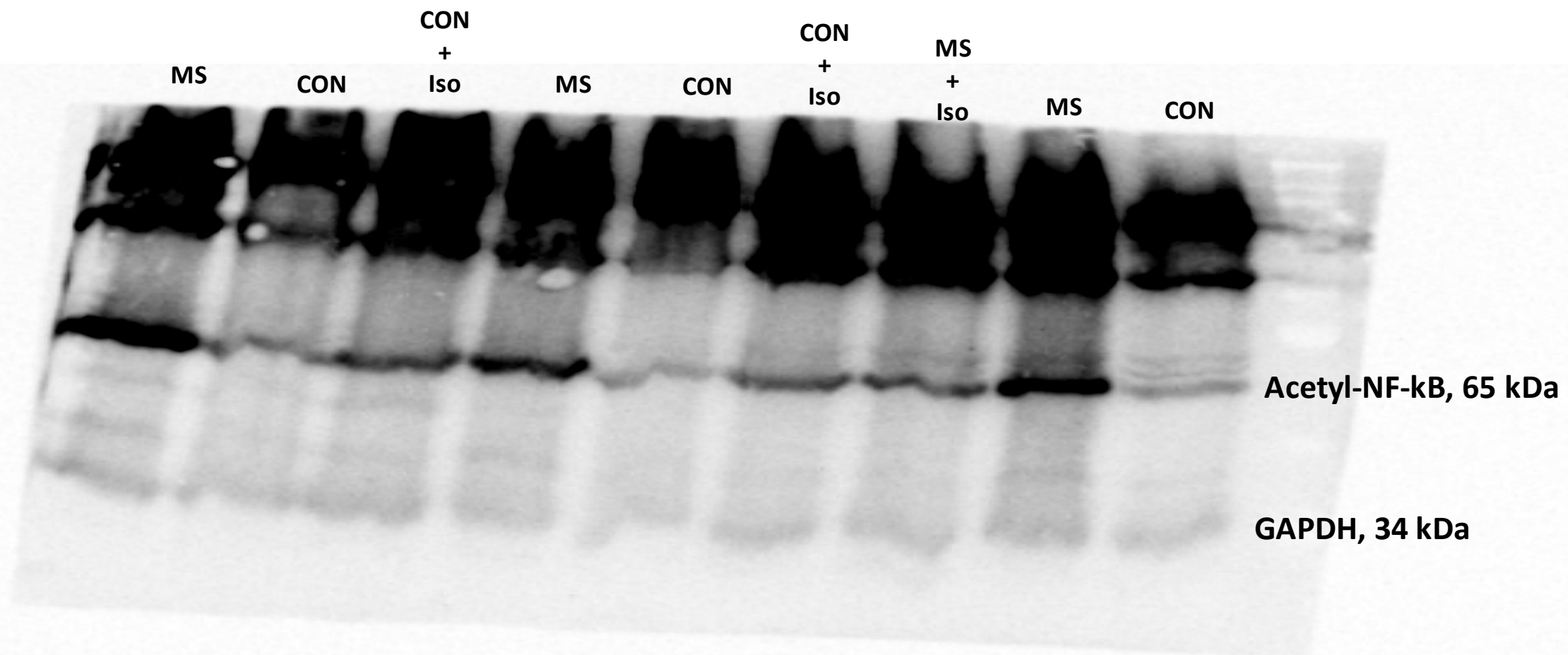

Supplement: Supplementary file 1 — (PDF 1.70 MB) [file 12035_2025_5483_MOESM1_ESM.pdf]
